# Viridot: An automated virus plaque (immunofocus) counter for the measurement of serological neutralizing responses with application to dengue virus

**DOI:** 10.1371/journal.pntd.0006862

**Published:** 2018-10-24

**Authors:** Leah C. Katzelnick, Ana Coello Escoto, Benjamin D. McElvany, Christian Chávez, Henrik Salje, Wensheng Luo, Isabel Rodriguez-Barraquer, Richard Jarman, Anna P. Durbin, Sean A. Diehl, Derek J. Smith, Stephen S. Whitehead, Derek A. T. Cummings

**Affiliations:** 1 Department of Biology, University of Florida, Gainesville, FL, United States; 2 Division of Infectious Diseases and Vaccinology, School of Public Health, University of California, Berkeley, Berkeley, CA, United States; 3 Department of Medicine-Infectious Disease, Vaccine Testing Center, University of Vermont Larner College of Medicine, Burlington, VT, United States; 4 Mathematical Modelling of Infectious Diseases Unit, Institut Pasteur, Paris, France; 5 Department of Epidemiology, Johns Hopkins Bloomberg School of Public Health, Baltimore, MD, United States; 6 Center for Immunization Research, Johns Hopkins Bloomberg School of Public Health, Baltimore, MD, United States; 7 School of Medicine, University of California, San Francisco, San Francisco, CA, United States; 8 Viral Diseases Branch, Walter Reed Army Institute of Research, Silver Spring, MD, United States; 9 Department of Zoology, University of Cambridge, Cambridge, United Kingdom; 10 National Institute of Allergy and Infectious Diseases, National Institutes of Health, Bethesda, MD, United States; 11 Emerging Pathogens Institute, University of Florida, Gainesville, FL, United States; Naval Medical Research Center, UNITED STATES

## Abstract

The gold-standard method for quantifying neutralizing antibody responses to many viruses, including dengue virus (DENV), is the plaque reduction neutralization test (PRNT, also called the immunofocus reduction neutralization test). The PRNT conducted on 96-well plates is high-throughput and requires a smaller volume of antiserum than on 6- or 24-well plates, but manual plaque counting is challenging and existing automated plaque counters are expensive or difficult to optimize. We have developed Viridot (Viridot package), a program for R with a user interface in shiny, that counts viral plaques of a variety of phenotypes, estimates neutralizing antibody titers, and performs other calculations of use to virologists. The Viridot plaque counter includes an automatic parameter identification mode (misses <10 plaques/well for 87% of diverse DENV strains [n = 1521]) and a mode that allows the user to fine-tune the parameters used for counting plaques. We compared standardized manual and Viridot plaque counting methods applied to the same wells by two analyses and found that Viridot plaque counts were as similar to the same analyst's manual count (Lin’s concordance correlation coefficient, ρ_c_ = 0.99 [95% confidence interval: 0.99–1.00]) as manual counts between analysts (ρ_c_ = 0.99 [95% CI: 0.98–0.99]). The average ratio of neutralizing antibody titers based on manual counted plaques to Viridot counted plaques was 1.05 (95% CI: 0.98–1.14), similar to the average ratio of antibody titers based on manual plaque counts by the two analysts (1.06 [95% CI: 0.84–1.34]). Across diverse DENV and ZIKV strains (n = 14), manual and Viridot plaque counts were mostly consistent (range of ρ_c_ = 0.74 to 1.00) and the average ratio of antibody titers based on manual and Viridot counted plaques was close to 1 (0.94 [0.86–1.02]). Thus, Viridot can be used for plaque counting and neutralizing antibody titer estimation of diverse DENV strains and potentially other viruses on 96-well plates as well as for formalization of plaque-counting rules for standardization across experiments and analysts.

## Author summary

Although the plaque reduction neutralization test (PRNT) is an important assay for measuring neutralizing antibody responses against many viruses, no free, open-source programs specifically designed for virus plaque counting and neutralizing antibody titer estimation are available. We have developed Viridot, a package for R with a user-interface in shiny, which is designed for use by laboratory-based virologists and researchers with minimal coding experience. The program includes: automatic and user-specification of settings for plaque counting; saving of plaque counting settings; counting of many plates at once; and easy output of plaque counts, plaque sizes, and images with counted plaques circled. Viridot also includes programs to analyze plaque counts, including estimation of: neutralizing antibody titers, pfu/mL of a virus stock, and the dilution factor of virus needed for an experiment. Viridot can be used to standardize plaque-counting methods within and between laboratories, helping researchers formalize an important aspect of the PRNT method that is often subjective. Viridot thus provides laboratory researchers around the world with a free tool to improve the speed and consistency with which the PRNT is conducted, aiding the public health response to emerging viral diseases.

## Introduction

The plaque reduction neutralization test (PRNT), developed in 1956 [1], remains the gold-standard method for measuring neutralizing antibodies against many pathogens. The PRNT was adapted for the study of dengue viruses 1–4 (DENV1-4) in 1967 [2] and has since been further adapted [3] and extensively used in dengue cohort studies [4–8]. The PRNT with immunostain-based detection of plaques is used as the primary assay for measuring immunogenicity of dengue vaccines and vaccine candidates [9–12]. The PRNT for DENV as recommended by the World Health Organization (WHO) consists of serial dilutions of antiserum incubated with a standard concentration of virus (40–60 plaque-forming units [pfu]) inoculated onto a cell monolayer on 6- or 24-well plates and incubated for 4–7 days to allow plaque growth [13]. Plaques are revealed either by applying a vital stain to cells (some reserve the term PRNT for this method) or by detecting plaques using a DENV-specific monoclonal antibody (some call this the immunofocus or focus reduction neutralization test, or FRNT) [13]. The terms PRNT and plaque are used here, consistent with the definition of ‘plaque’ provided in Fields Virology as a lytic or non-lytic ‘area of infection’. Despite common usage, we do not use the term 'focus', defined in Fields Virology as 'a pile of transformed cells' caused by infection with tumor-causing virus, as this is not an accurate description of DENV infection [14]. WHO recommends counting DENV plaques manually [13], generally directly from the plate or under a microscope. It is desirable to use 96-well plates for the PRNT, which require smaller volumes of antiserum and fewer days for plaque growth (2–4 days). However, to achieve the target number of pfu/well, plaques must be smaller to prevent plaque overlap [3] and thus are more difficult to count manually.

Automated counters for immunostained plaques do exist, but current versions also present multiple challenges. With commercial plaque counting software such as the ImmunoSpot and Biospot software on CTL Analyzers (Cellular Technology Ltd.), researchers typically optimize a small panel of strains for plaque intensity and size and develop programs or macros to count plaques for each of these strains [15,16]. However, for researchers working with diverse strains with varied plaque phenotypes, such programs are often difficult to optimize and standardize. Additionally, commercial counters are often proprietary and expensive. Free, open-source general image processing tools are available that allow for plaque counting on a personal computer, such as ImageJ [17], but these programs require users to navigate through a series of menu bars to identify relevant image processing tools or write their own macros; further, as the code is written in JavaScript, many researchers may struggle to adapt image-processing code beyond what is available. To our knowledge, there are not programs that include integrated plaque counting and neutralization titer estimation functionality.

We have designed Viridot (Viridot package), a free, open-source, automated package in R [18] for immunostained viral plaque counting and neutralizing antibody titer estimation with a user interface in shiny [19] and image processing tools from the EBImage package [20]. The R statistical framework is open source statistical software with a large community of users that contribute packages enabling a multitude of specific functions. Viridot is designed to be relatively simple to set up and learn for new users without coding experience as well as easy to optimize to each assay. Among other features, Viridot includes plaque counting with an automatic parameter identification mode; a single interface that enables easy access to all modifiable parameters at once; tools to break down each image-processing step to aid in the optimization process; counting of single or multiple plates; and the ability to save images with circled plaques as well datasets of plaque sizes, parameter settings, and plaque counts in 96- or 24-well plate format. The Viridot plaque counter can be used for images acquired with different light settings as well as spots of different stain colors. Viridot also has a built-in neutralizing antibody titer estimation program with many modifiable parameters and programs for estimating virus concentrations from virus titration assays as well as the dilution factor to achieve the correct number of pfu/well in a neutralization test.

Here, we describe the Viridot programs, explain the process for standardizing manual and Viridot plaque counting methods, and evaluate the performance of Viridot on a variety of plaque images. We show that for the same set of wells, a single analyst can achieve plaque counts with Viridot that are as similar to their own manual count as to the manual count of a different analyst. Further, we show that neutralizing antibody titers estimated from Viridot plaque counts are highly similar to those estimated from manual counts.

## Methods

### Ethics statement

This study followed the National Institutes of Health guidelines for the humane treatment of laboratory animals and was approved by the NIAID Animal Care and Use Committee (11DEN33 and 14DEN34, parent protocol NIAID ASP LID 9). Human sera from NIH monovalent and tetravalent DENV vaccines trials (ClinicalTrials.gov identifiers: NCT00473135, NCT00920517, NCT00831012, NCT01072786) were performed under an investigational new drug application reviewed by the U.S. Food and Drug Administration and approved by the Institutional Review Board at the University of Vermont and Johns Hopkins University. Johns Hopkins Bloomberg School of Public Health approved the study and collection of a DENV-positive human antiserum and coordinated the IRB review conducted by Western Institutional Review Board. Additional human sera were collected from the NIAID vaccine study for Tick-Borne Encephalitis Virus study (ClinicalTrials.gov identifier NCT01031537). In all studies, informed written consent was obtained in accordance federal and international regulations (21CFR50, ICHE6).

### Viridot development and distribution

Viridot was written in R 3.3.2 (R Foundation for Statistical Computing [18]). We used shiny [19] and shinyFiles [21] for creating the user interface and the EBImage package [20] for many of the image processing tools. Viridot is available on GitHub at: https://github.com/leahkatzelnick/Viridot. Installation instructions for Mac and Windows are available on the GitHub webpage and in the Viridot manual (S1 Supporting File).

### Importing and exporting files and data

Through the shiny interface the user can load previous parameter settings and save current parameter settings (mixedsort and file_ext from gtools [22]). The user is able to analyze images from multiple plates, subsets of wells on a plate, or single wells (jpeg, jpg, png, tiff, tif, and CTL files can be analyzed). After running the plaque counter, the user can print images with circled plaques (paintObjects [20]), tables of plaque counts organized in 24- or 96-well plate format or as a list, and tables of plaque radius or area (computeFeatures.shape [20]) for each well.

### Image processing pipeline

The user has the option of seeing how the image data is affected by the parameter settings for each image-processing step with the *Show what is done at each step* option as part of the optimization process (S1 Fig). The image-processing steps are as follows. The Viridot plaque-counting program analyzes pixel intensity values ranging from 0–1, requiring conversion of the raw image data into a single channel that can be analyzed as a grayscale image. The amount and type of light used for taking the well image determines the optimal color channel that provides the most information on the difference between background (should be closer to values of 0, or dark) and plaque (closer to values of 1, or light). The user can select the red, green, or blue channels, hue, saturation, or value channels (rgb2hsv function in the grDevices package), or a combination of these channels. Images can then be smoothed to reduce noise by applying a Gaussian blur (gblur, EBImage package [20]) with a sigma value (radius in pixels for the Gaussian transformation) defined by the user. An extra option allows removal of strings or fibers in image that are a different color from plaques. Because well edges may contain reflections, excess liquid, and other distortions that can interfere with the plaque counting, images can be trimmed so that only pixels more than a certain distance from the well edge are included in the analyses. To increase the difference between background and plaque, pixel data can be transformed by multiplying and/or exponentiating the image pixel data by constants, ensuring that the subsequent threshold step is applied to the pixel difference between background and plaque, and not in some other minimum in pixel intensities. A thresholding function with a square thresholding filter (makeBrush and thresh [20]) is used to analyze the transformed image to identify plaque shapes. Plaque shapes from the thresholding step can be dilated (dilate [20]) to consolidate plaques that may be broken into multiple small objects. Distinct plaques that are touching can be divided using a watershed function (distmap and watershed [20]), with user-specified tolerance for touching objects. Finally, the user specifies the minimum and maximum size of an object (total number of pixels per object) that should be counted as a plaque.

### Automatic parameter-identification mode

The Viridot automatic parameter identification mode analyzes the image for each well to identify parameters that best distinguish plaques from background for that well. Darkness and size of the plaque (pixels with intensity>0.8)/(pixels with intensity>0.5), and the amount of background *(*peak in the histogram of pixel intensities), are estimated. These parameters are used to identify parameter settings for the Gaussian blur, image transformation, and thresholding (See Viridot manual in S1 Supporting File).

### Neutralizing antibody titer estimation

The neutralizing antibody titer estimation program creates dose-response curves (2- or 3-parameter logistic regression with drm in drc package [23]) from a table of plaque counts based on a plate template specifying the positions of samples, dilutions, and controls as defined by the user (Viridot manual in S1 Supporting File). The user can select from multiple methods for estimating neutralization titers, write in sample and experiment names, define the dilution series, designate the PRNT titer cut-off (PRNT_10_ –PRNT_90_), or run only single titrations at once. Control well plaque counts can be inserted manually or estimated from specified wells on the plate. The program outputs the PRNT titers with 95% confidence intervals, the curve slope, and a plot showing the neutralization curves with 95% confidence intervals, with customizable color schemes and methods for plotting error bars of the raw data. A program similar to the LID Statistical Web Tools Plaque Reduction tab (https://exon.niaid.nih.gov/plaquereduction/) is also available for PRNT titer estimation.

### Other Viridot tools

Tools for estimating the virus dilution required for neutralization tests (e.g. 40 pfu/well) and a tool for estimating virus titer (pfu/mL) from a virus titration are also included in the Viridot package.

### Image formatting

This tab allows the user to cut an image of multiple wells into individual square well images, enabling users to more easily use camera phones for well image acquisition. The user can also trim individual well images to square images.

### Standardized rules for manual and Viridot plaque counting

Standardized manual plaque counting rules were established by having four analysts discuss the plaque counting process for a well of plaques with uniform size and intensity (S2A Fig) as well as non-uniform size and intensity (S2B Fig). The following rules were established. (1) All spots either totally or partially within the specified well area were counted. (2) Spots more than approximately twice the size of the average dark spot were divided using reasonable break-points. (3) Pale spots about the size of the average spot were counted as plaques. 4) Spots less than approximately half the size of the average spot and non-blue artifacts (e.g. strings or fibers) were not counted.

We also developed rules for optimizing plaque-counting parameters for Viridot. (1) Run the automatic parameter identification mode with plaques counted within circle ≥25 pixels from the image edge. (2) Identify the well on the plate expected to be most poorly counted by the plaque counter, and modify the following parameter settings (in the user-adjustable mode), in this order: (i) Gaussian blur (decreased), (ii) contrast, (iii) threshold, (iv) dividing overlapping plaques, (v) minimum plaque size (increased), and (vi) dilation (decreased). (3) Once a satisfactory count was achieved, the user assessed three additional random wells to check the final quality of the plaque count and make any additional adjustments. The final parameter settings were then applied to count plaques for the full plate.

### Data sets

The automatic-parameter identification mode training set and test set 1 each consisted of 1521 well images of PRNT titrations conducted on C6/36 cells (*Aedes albopictus*, NIAID/Novavax MCB) (full PRNT protocol in S2 Supporting File). The PRNT titrations were of 47 genetically diverse DENV1-4 strains (GenBank accession numbers KT382187, KT452791, GQ868619, FJ639680, AY145121, AY780643, EU482591, AF180817, KT382186, FJ461335, KT382189, KT452795, GU131927, GQ868638, KT382188, FJ538920, FJ467493, AF038403, EU482756, EU482684, AY158339, EF105384, AY744147, KT452797, KT452796, EU482672, KT452792, KT452799, L11422, KT452798, HQ541806, KT452800, EU529698, EU660409, FJ432743, KT452794, KT452793, KF543272, KT452802, AF326573, JN022608, KT452801, JF262780, KT452803, EU854297, FJ882599, and AY780644) titrated against de-identified human biorepository samples from prior clinical trials of monovalent components of a live attenuated dengue vaccine [24] or African green monkey (*Chlorocebus sabaeus*, NHP) antisera [25]. The training set consisted of well E7 from each plate; test set 1 was well H9. Test set 2 consisted of 421 additional well images randomly drawn from the control antiserum titration plate for PRNT titrations of the African green monkey antisera against 107 Thai DENV1-4 strains (subset of strains in [26], GenBank accession numbers KY586306 to KY586946) that had not been used in the training set or in test set 1. The manual plaque-counting methods comparison (n = 72 wells) and Viridot plaque-counting methods comparison (n = 32 wells) used 96-well plates of C6/36 cells inoculated with a DENV3 strain (GenBank accession number HQ541806) at a target of 40 pfu/well. Comparison of standardized manual and Viridot plaque-counting methods consisted of 168 wells of the DENV3 strain titrated against DENV-positive antiserum (three titrations on the same plate, three titrations on different plates in different experiments). Plaque data from titrations of African green monkey antisera against a subset of the DENV1-4 strains on C6/36 cells (n = 240 wells [24,25]) were also studied. All plaque data were stained with peroxidase-labeled antibody and TrueBlue peroxidase substrate (full details in S2 Supporting File). Plates were scanned with the ImmunoSpot Analyzer (CTL, Shaker Heights, OH), with a light source from above using the standard Costar 96 well template for clear plates (details about the plate, camera, and light settings are provided in S1 Table). Well images were saved as .CTL images (encrypted .tiff files) of 752 by 752 pixels.

A second laboratory used human sera (de-identified samples from a prior clinical trial of a live attenuated dengue vaccine [27] or human monoclonal antibodies [28]) against a DENV2 strain (AF038403) and ZIKV strain (KX280026) on Vero (African green monkey kidney) cells (n = 160). Plaques were stained with peroxidase-labeled antibody and TrueBlue peroxidase substrate (S2 Supporting File). All plates were scanned using a Zeiss Axio Imager.M1 microscope with a 2.5X lens and the AxioCam MRc mounted camera controlled by the software KSElispot (Windows 95). Well images were saved as .TIF images of 1040 by 1040 pixels.

We also imaged plates from the top (well-side) and the bottom of 96 well plates using camera phones. A set of 45 well images from clear plates (placed on white paper) were taken using the iPhone 7 plus version 11.4.1, model MN5L2LL/A, without flash. The camera phone was balanced on a plastic test tube holder 9cm above the plate, with bench lighting 39 cm above plate. Five additional well images taken from the top of white 96 well plates were acquired using the Samsung Galaxy S7, model SM-G930V, also without flash. Camera phone images were divided into individual wells using the crop function in Preview and trimmed to square images using the Viridot image formatting program. Images ranged in size but were a minimum of 200 by 200 pixels to ensure sufficient resolution for counting.

A small panel of wells that captured a more diverse array of light and staining methods were also analyzed. A well of ZIKV plaques on Vero cells was acquired using the Zeiss Axio Imager.M1 microscope with a 2.5X lens and the AxioCam MRc mounted camera, using bright top lighting. A well of IFN-γ T-cell responses to Influenza A nucleoprotein (NP) peptide _147–155_ was measured using an IFN-γ specific biotinylated monoclonal antibody stained with phosphate-labeled streptavidin and 5-bromo, 4-chloro, 3-indolylphosphate/nitroblue tetrazolium substrate [29,30]. The image was taken with Zeiss Axio Cam Imager.M2 scope with the AxioCam MRc camera, 5x objective lens (image courtesy of J. Misplon). A well of the total and DENV4-specific IgG+ memory B cells were from an ELISPOT-based Multi-Color FluoroSpot assay [31]. Spots were detected by adding either a TRITC-labeled IgG-specific antibody or antigen of each serotype and virus-specific antibodies each labeled with a fluorescent Qdot of a different color. Images were acquired using the ImmunoSpot Analyzer (images courtesy of P. Andrade). Wells of plaques of recombinant lymphocytic choriomeningitis virus (LMCV) on Vero E6 cells were resolved by applying crystal violet and ethanol in water [32]. Plates were imaged using Alpha Innotech digital camera with the Computar H6Z0812M motorized zoom lens and divided into individual well images using the Viridot image formatting program (images courtesy of C. Ziegler).

### Statistical analyses

The Viridot automatic parameter identification mode was evaluated by having an analyst classify counted well images by number of missing plaques in the following ranges: 0, 1–3, 4–7, 8–14,15–19, 20–30, >30. Absolute number of plaques missed and the proportion of plaques missed (automatic count/[plaques missed + automatic count]) for the training and test sets are presented as pie charts.

For comparisons of manual and Viridot counts, exact numbers of plaques were counted. Agreement between analysts for each method or for a given analyst between methods was estimated with Lin’s concordance correlation coefficient, ρ_c_, a measure of the extent to which observations deviate from a line with slope 1, intercept 0 (45° line through origin) with ρ_c_=1 indicating perfect concordance, -1 perfect discordance, and 0 no concordance (values ≥90% indicate good concordance). We also estimated the ratio of plaque counts by different methods to enable evaluation of bias toward overcounting by Viridot (e.g., Manual:Viridot ratio less than 1) or undercounting by Viridot (e.g., Manual:Viridot ratio greater than 1). For both measures, 95% confidence intervals are shown (95%CI). We also estimated the ratio of PRNT_50_ titers with 95% confidence intervals for titrations with plaques counted by different methods. PRNT_50_ titers were estimated without allowing for a resistant fraction, without using the LID Statistical Web Tools method, and using the 'eachtop' setting for defining plaque counts in the absence of neutralization. To summarize results across experiments, we present geometric averages of the ratios of plaque counts as well as PRNT_50_ titers; ratios that include 1 in the confidence interval indicate there is not a significant difference between methods.

## Results

**Fig 1:**
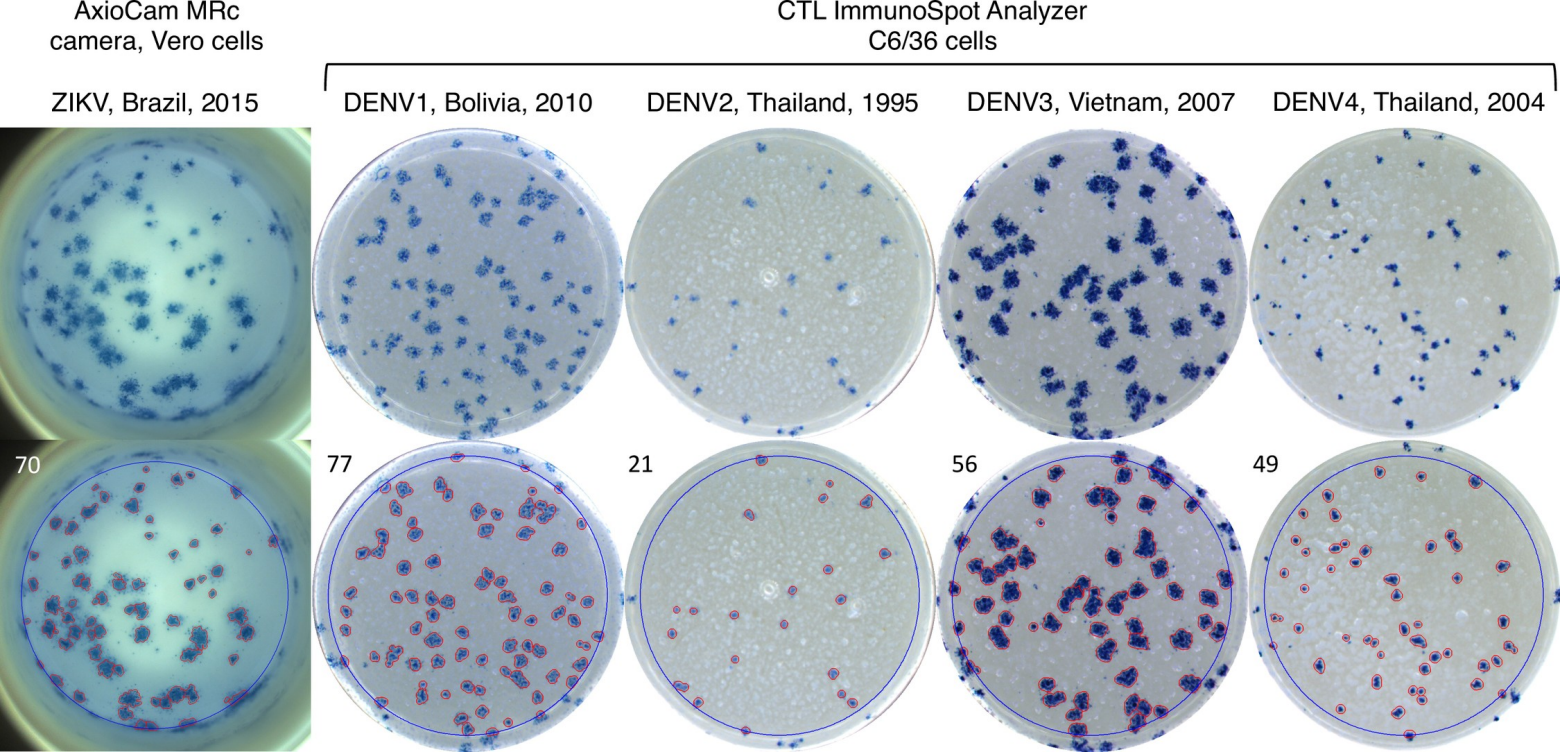
Example well images of DENV and ZIKV plaques and resulting Viridot plaques counts. The type of camera used for acquiring the image, cell type for infection, and the virus (with location and year of isolation) is printed above each raw image. The image in which plaques have been counted is shown below, with the resulting plaque count printed in the top left corner. Well images reflect separate experiments done by different analysts.

Viridot was developed using PRNT data of genetically diverse DENV strains in >100,000 plaque well images from experiments conducted over many years. Most well images were of plaques on clear 96 well plates immunostained with peroxidase-labeled antibodies with peroxide substrate TrueBlue. Plates were imaged with CTL ImmunoSpot Analyzer and saved as square .TIF and .JPG images of more than 500 by 500 pixels. Examples of well images that capture the range of plaque phenotypes used for developing Viridot and optimizing the automatic parameter identification mode, along with corresponding images of counted plaques, are shown in Fig 1.

**Fig 2:**
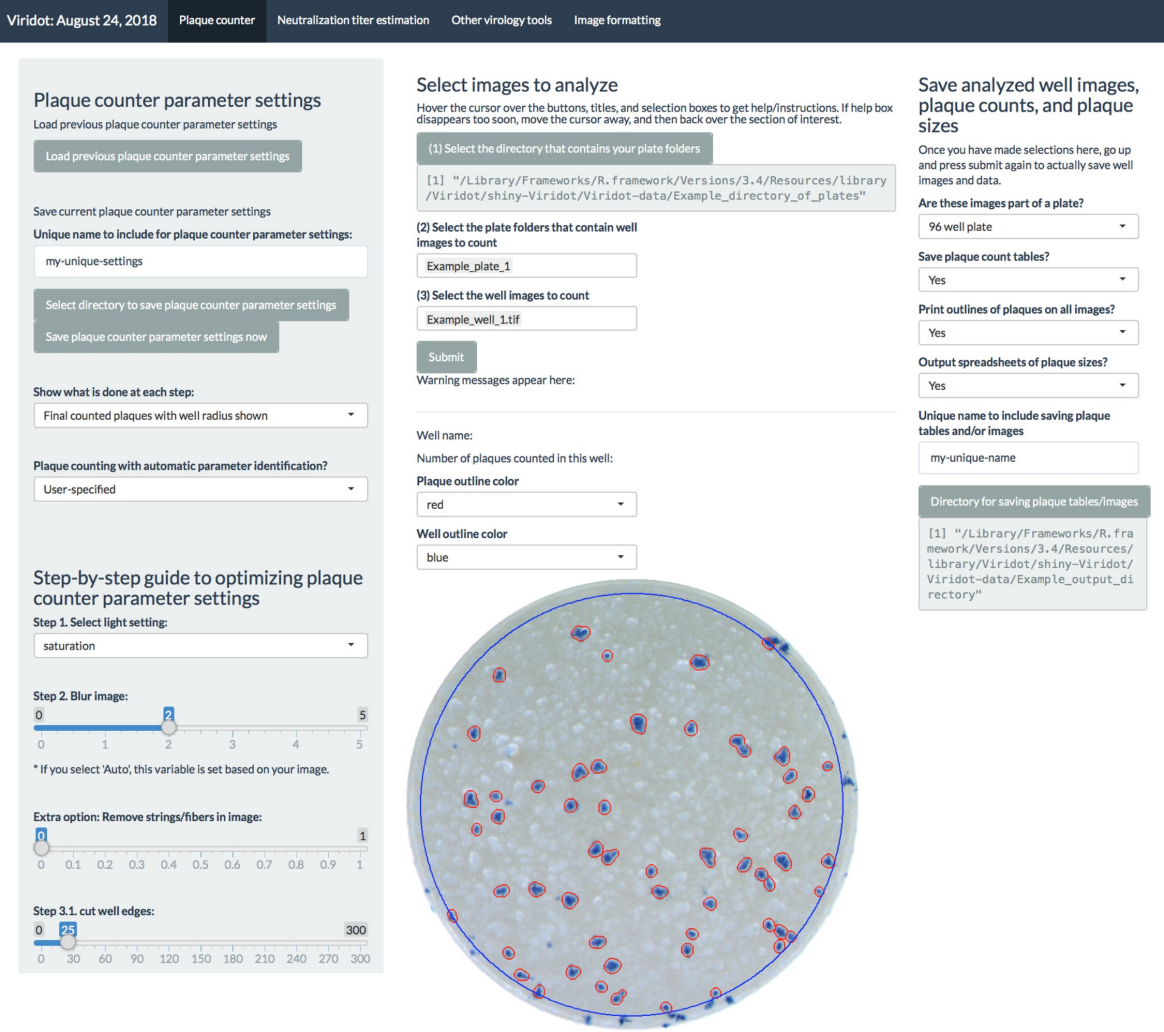
The Viridot plaque counter program. The scrollable panel on the left contains the parameter settings for plaque counting and buttons to load and save parameter settings. Selection of plates and images for analysis is shown in the center. A well containing DENV plaques is shown, with counted plaques circled in red and the well area in which plaques were counted circled in blue. The plaque count is shown above the image. The panel to the right includes options to save plaque count tables, circled plaque images, and plaque sizes.

To enable the user to optimize the Viridot plaque counting program (Fig 2) to count diverse virus plaque phenotypes, we incorporate two main features: 1) an automatic parameter identification mode to help the user have the best ‘first pass’ parameters for counting particular well images and 2) user adjustment of parameter settings, with the option of seeing the effect of each step in the image processing pipeline, to enable the user to fine-tune parameters for that particular virus or experiment.

### Evaluation of the automatic Viridot plaque counter parameter-identification mode

**Fig 3:**
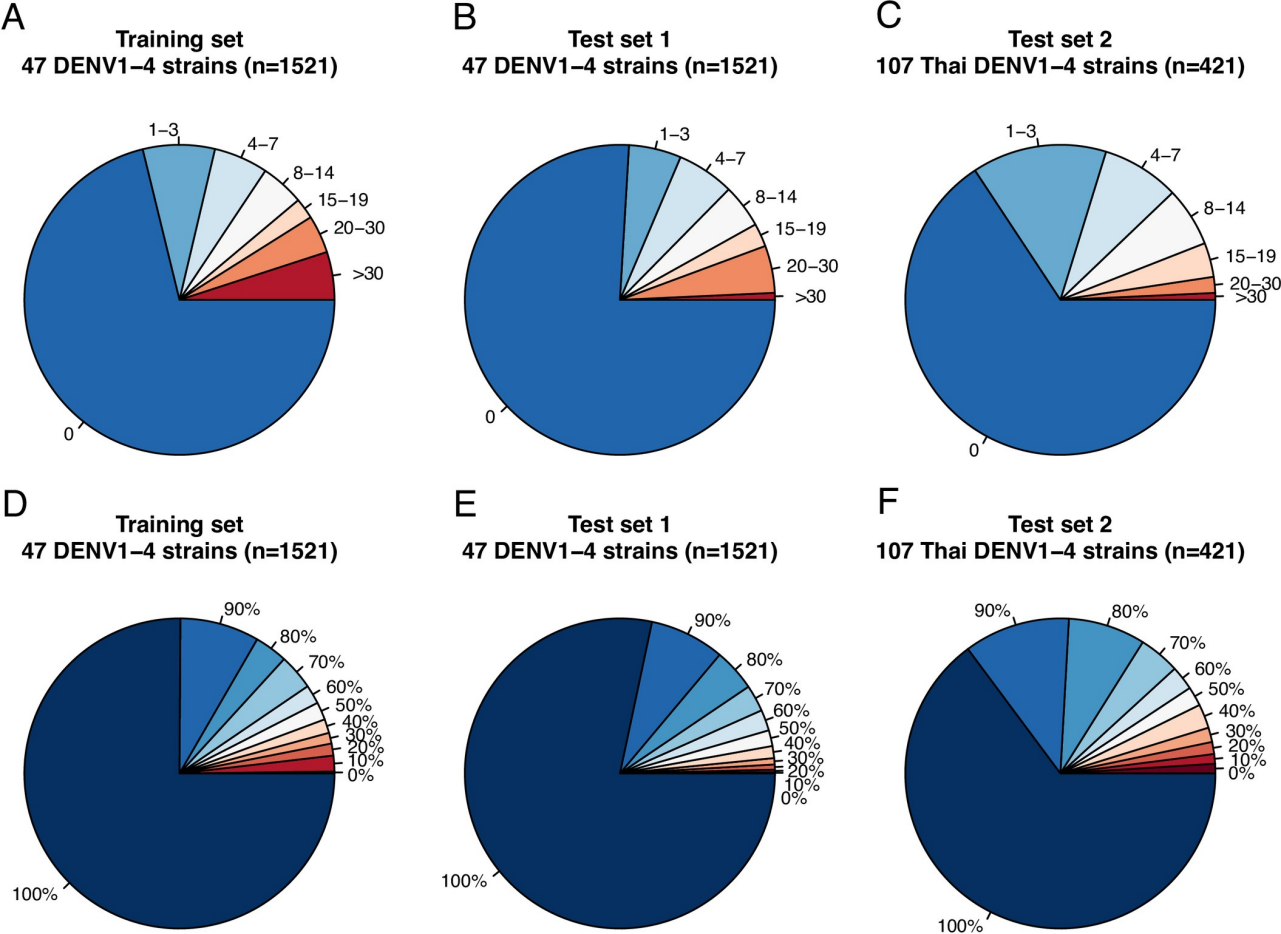
Evaluation of the Viridot automatic parameter-identification mode for a training set and two test sets. Total number **(A-C)** or proportion **(D-F)** of plaques per well image counted by the Viridot automatic parameter-identification program that are discordant with analyst-estimated plaque counts. A single analyst estimated the number of miscounted plaques by checking raw images for plaques not circled by the automatic counter.

The automatic parameter-identification mode was developed using a training set (n = 1521 well images) of plaques of genetically diverse DENV1-4 strains. The automatic mode missed <10 plaques/well for 84% of the training set and achieved a plaque count ≥80% of the analyst-classified count for 86% of the training set (Fig 3). The quality of the Viridot automatic program was evaluated for a different randomly selected well from the same 1521 plates (test set 1) and for 421 additional well images of plaques from 107 DENV strains not used in the training set (test set 2). For test set 1 and 2, 87% and 87%, respectively, of automatically-counted wells missed <10 plaques and 90% and 83%, respectively, of wells had ≥80% the number of plaques of the analyst-classified count (Fig 3).

### Standardization of manual and Viridot parameter adjustment methods for plaque counting

Two analysts (designated A1 and A2) each counted the same 72 wells of DENV3 plaques directly from the plate, under a microscope, and from the printed image of the well (752 by 752 pixels) with a circle of constant radius within which plaques were to be counted. We measured within and between analyst variation in plaque counting using ρ_c_, which measures deviation from perfect concordance, as well as ratio of plaque counts (Table 1). For both A1 and A2, within-analyst concordance was poor across methods (ρ_c_<0.67 for all comparisons) but A1 and A2 had better agreement for plaque counts from the printed well image (ρ_c_ = 0.81 [95% CI: 0.72–0.87], ratio of plaque counts, 0.96 [95% CI: 0.94–0.98]). Based on these observations, we developed standardized plaque counting rules by seeking consensus among four analysts about what did and did not qualify as a plaque (see Methods).

**Table 1:** Comparison of manual plaque counting methods within and between Analyst 1 and Analyst 2 (n = 72 wells).

**Within Analyst** **metdods comparison**	**Analyst 1**		**Analyst 2**	
	*Concordance ρ*_*c*_ *(95% CI)*	*Plaque count ratio (95% CI)*	*Concordance ρ*_*c*_ *(95% CI)*	*Plaque count ratio (95% CI)*
From plate vs. under microscope	0.67 [0.53–0.78]	1.00 [0.96–1.03]	0.07 [0.01–0.13]	0.73 [0.7–0.76]
From plate vs. printed image	0.60 [0.44–0.72]	0.94 [0.91–0.97]	0.06 [-0.12–0.25]	0.90 [0.86–0.94]
Under microscope vs. printed image	0.62 [0.47–0.74]	0.94 [0.91–0.97]	0.15 [0.06–0.24]	1.23 [1.2–1.27]
**Between Analysts methods comparison**	**Analyst 1 vs. Analyst 2**			
	*Concordance ρ*_*c*_ *(95% CI)*	*Plaque count ratio (95% CI)*		
From plate	0.19 [-0.04–0.4]	0.99 [0.95–1.05]		
Under microscope	0.14 [0.07–0.2]	0.73 [0.70–0.76]		
Printed image	0.81 [0.72–0.87]	0.96 [0.94–0.98]		

Deviation from perfect concordance of 1 between methods (ρ_c_, Lin's concordance correlation coefficient) and geometric average ratio of plaque counts by different methods, shown with 95% confidence intervals (95% CI).

We also evaluated within- and between-analyst differences in Viridot plaque counting methods for an additional 36 wells of DENV3 plaques, comparing the automatic parameter-identification and the user-adjusted parameter mode, with parameters adjusted to the same well or wells chosen by each analyst separately (Table 2). There was good agreement between A1 and A2 counts for the user-defined settings adjusted to the same well (0.87 [95%CI: 0.81–0.91]), and as expected, perfect concordance for the automatic parameter identification mode. Based on these observations, we developed a standardized protocol for optimizing user-specified settings for the Viridot counter (see Methods).

**Table 2:** Comparison of Viridot plaque counting methods within and between Analyst 1 and Analyst 2 (n = 36 wells).

**Within Analyst** **methods comparison**	**Analyst 1**		**Analyst 2**	
	*Concordance ρ*_*c*_ *(95% CI)*	*Plaque count ratio (95% CI)*	*Concordance ρ*_*c*_ *(95% CI)*	*Plaque count ratio (95% CI)*
Auto vs. user-specified, same well	0.28 [0.15–0.39]	0.83 [0.79–0.86]	0.51 [0.39–0.61]	0.85 [0.82–0.89]
Auto vs. user-specified, any well	0.12 [0.02–0.22]	0.77 [0.73–0.81]	0.92 [0.88–0.94]	0.93 [0.91–0.94]
User-specified, same well vs. any well	0.82 [0.76–0.86]	0.93 [0.92–0.94]	0.69 [0.59–0.77]	1.08 [1.06–1.11]
**Between Analysts methods comparison**	**Analyst 1 vs. Analyst 2**			
	*Concordance ρ*_*c*_ *(95% CI)*	*Plaque count ratio (95% CI)*		
Auto	1	1		
User-specified, same well	0.87 [0.81–0.91]	1.03 [1.02–1.05]		
User-specified, any well	0.22 [0.09–0.34]	1.21 [1.16–1.26]		

Deviation from perfect concordance of 1 between methods (ρ_c_, Lin's concordance correlation coefficient) and geometric average ratio of plaque counts by different methods, shown with 95% confidence intervals (95% CI).

### Comparison of standardized manual and Viridot plaque counts and neutralizing antibody titers

**Fig 4:**
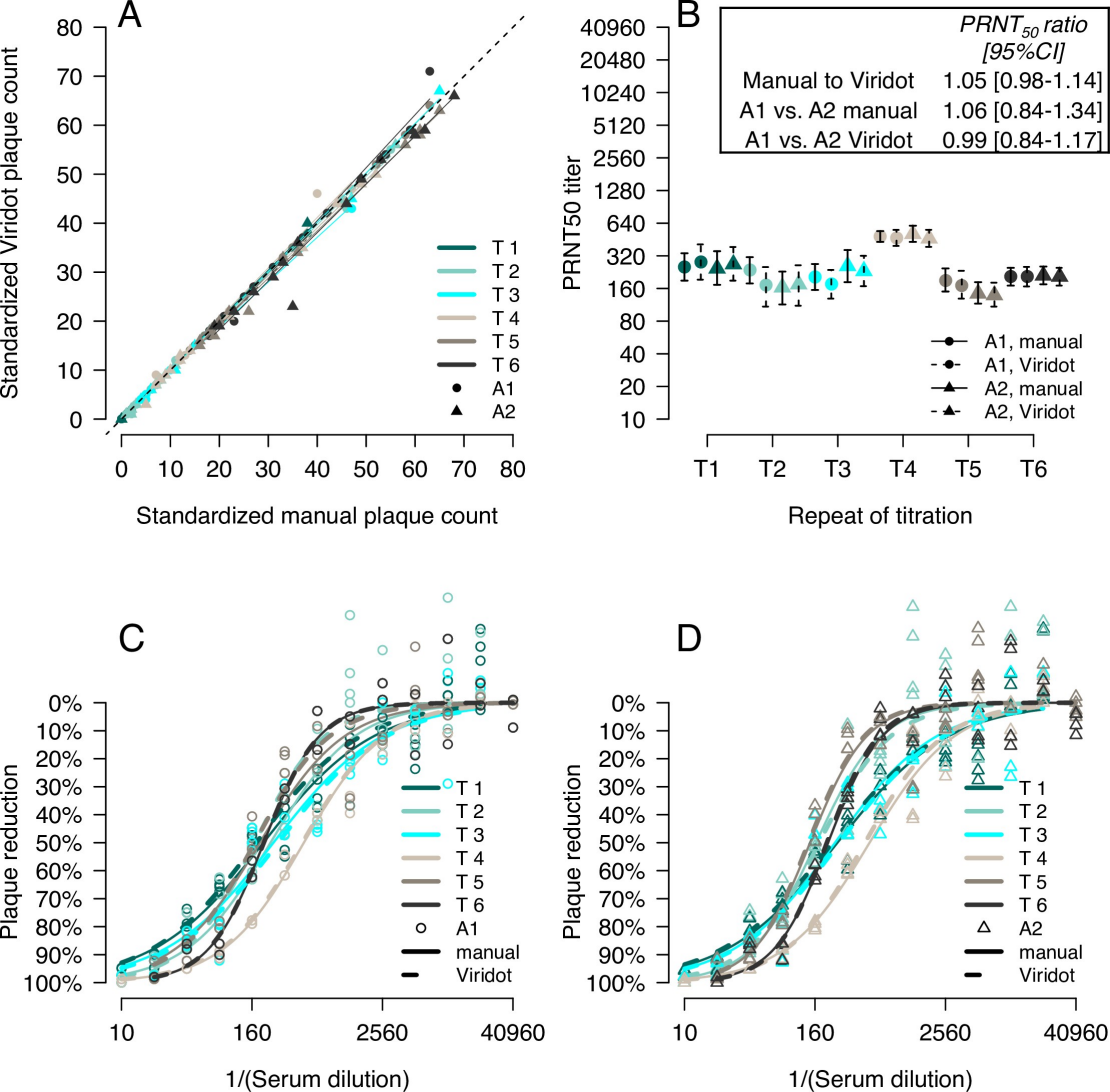
Comparison of standardized manual and Viridot plaque counts and neutralizing antibody titers. **(A)** Comparison of manual and Viridot counts of DENV3 plaques on C6/36 cells for six repeat titrations of a DENV3-positive antiserum by two analysts (A1 and A2). Linear regression lines are shown for each titration and analyst. The grey line indicates perfect correspondence. **(B)** PRNT_50_ titers with 95% confidence intervals for the six titrations counted by A1 and A2, manually and with Viridot. Resulting neutralization curves for A1 **(C)** and A2 **(D)** for the six titrations, counted either manually (solid lines) or with Viridot (dotted).

With these standardized manual and Viridot counting methods, we compared counts within and between analysts for an additional 168 wells of DENV3 plaques on C6/36 cells, consisting of six titrations of a DENV-positive antiserum (three from the same plate and experiment, three each from different plates and experiments, Tables 3 and S2 and Fig 4A). Within analyst, manual and Viridot counts were strongly in agreement (ρ_c_: A1 = 0.99, A2 = 1.00) with a slight tendency of Viridot to undercount plaques (ratio of manual to Viridot counts: A1 = 1.02, A2 = 1.04), although not significantly (Table 3). Between analysts, there was good agreement for both the Viridot method (ρ_c_ = 0.99, ratio of plaque counts = 0.97) and the manual counting methods (ρ_c_ = 0.99, ratio of plaque counts = 0.95, Table 3).

**Table 3:** Comparison of the standardized manual and Viridot plaque counting methods within and between Analyst 1 and Analyst 2 (n = 168 wells).

**Within Analyst** **methods comparison**	**Analyst 1**		**Analyst 2**	
	*Concordance ρ*_*c*_ *(95% CI)*	*Plaque count ratio (95% CI)*	*Concordance ρ*_*c*_ *(95% CI)*	*Plaque count ratio (95% CI)*
Manual vs. Viridot	0.99 [0.99–1.00]	1.02 [1.01–1.03]	0.99 [0.99–1.00]	1.04 [1.03–1.05]
**Between Analysts methods comparison**	**Analyst 1 vs. Analyst 2**			
	*Concordance ρ*_*c*_ *(95% CI)*	*Plaque count ratio (95% CI)*		
Manual	0.99 [0.98–0.99]	0.95 [0.94–0.97]		
Viridot	0.99 [0.99–0.99]	0.97 [0.96–0.98]		

Deviation from perfect concordance of 1 between methods (ρ_c_, Lin's concordance correlation coefficient) and geometric average ratio of plaque counts by different methods, shown with 95% confidence intervals (95% CI).

**Fig 5:**
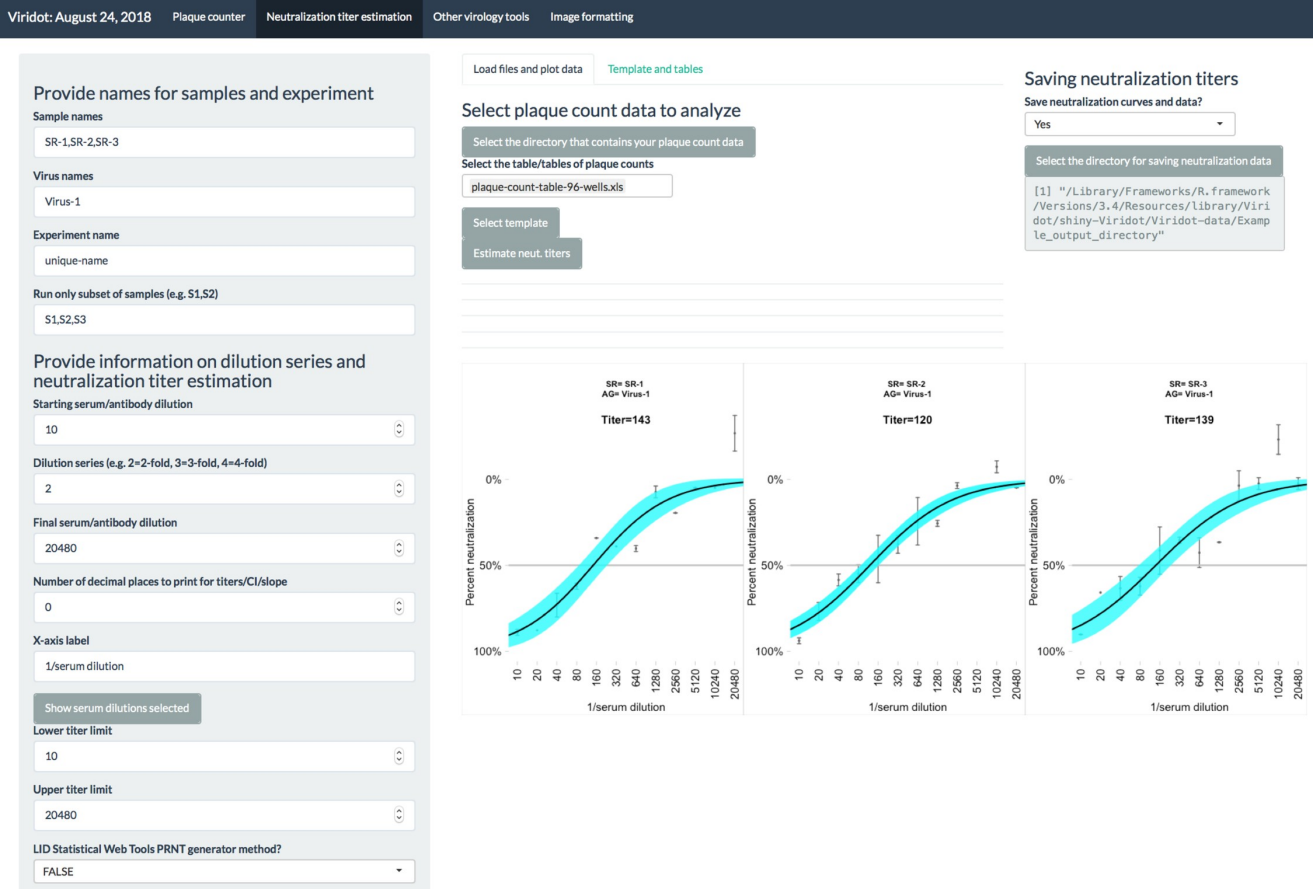
The Viridot neutralization titer estimation program. The scrollable panel on the left contains the parameter settings for neutralizing antibody titer estimation. In the top-center, users can import plaque count tables and the plate template (the user can switch tabs to see the imported templates and tables). In the center, summary statistics for control wells and the neutralizing antibody titration curves are shown, with 95% confidence intervals in blue and error bars (± 1sd shown) in gray. The estimated neutralizing antibody titer is printed above. The panel to the right includes options to save neutralizing antibody titer data to a table and to print the image of neutralization curves.

We also measured the consistency of neutralizing antibody titers (Fig 4B) within and between experiments for the two analysts using the standardized manual and Viridot counted plaque data. Neutralizing antibody titers were estimated for each of the plaque counting methods using the Viridot neutralization titer estimation tool (Fig 5). Overall, the geometric average ratio of the PRNT_50_ for the same titration between manual and Viridot counted plaques was 1.05 [95%CI: 0.98–1.14], indicating manual counted titrations had slightly higher PRNT_50_ titers, although not significantly. The average ratio of A1 to A2 manual counted antibody titers was 1.06 [0.84–1.34], similar to the average ratio of A1 to A2 Viridot counted antibody titers (0.99 [0.84–1.17]). Both manual counts and Viridot counts produced neutralizing antibody titers with 95% confidence intervals of less than a two-fold difference, with slightly narrower confidence intervals for manual counted titrations (manual count = 0.73 ± 0.22 [log_2_ scale], Viridot = 0.84 ± 0.27). Neutralization curves for manual counts were closer to the Viridot counts of the same titration than to manual counts of other titrations (Fig 4B–4D).

### Manual and Viridot plaque counts and neutralizing antibody titers for diverse flavivirus strains and antibody samples

**Fig 6:**
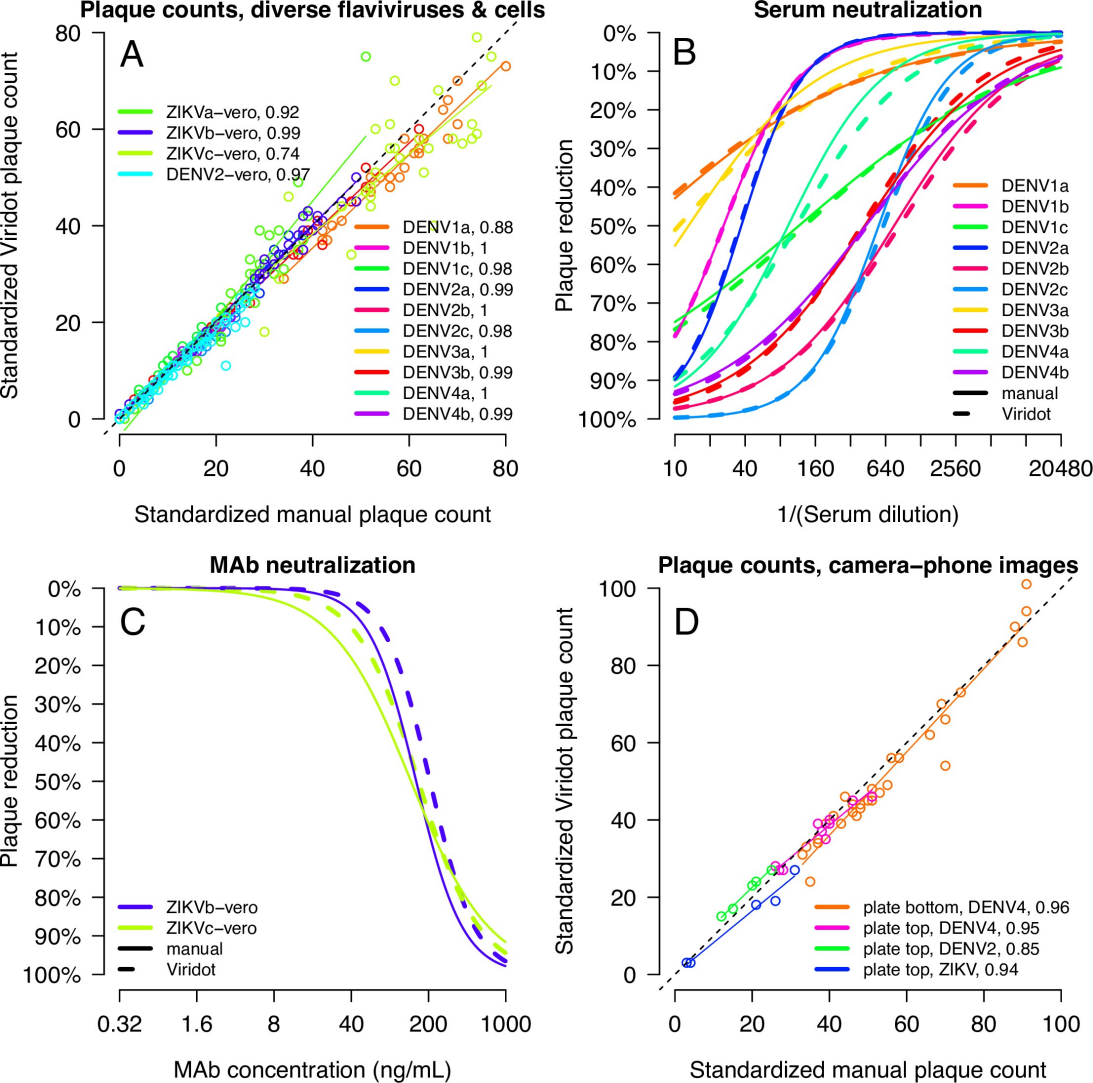
Comparison of manual and Viridot counts of DENV1-4 and ZIKV plaques and neutralizing antibody titers on C6/36 cells or Vero cells. **(A)** Comparison of standardized manual and Viridot plaque counts, with linear regression (solid lines) showing concordance: ρ_c_ values for each titration are indicated in the legend. The dotted black line indicates perfect correspondence. **(B and C)** Resulting neutralization curves for each antiserum titration, counted either manually (solid lines) or with Viridot (dotted). The geometric average ratio of the manual to Viridot for all PRNT_50_ titers is 0.94 (95% CI: 0.86–1.02). **(D)** Comparison of standardized manual and Viridot counted plaques in well images acquired using camera phones. Linear regression lines (solid lines), concordance (ρ_c_) values for plaque data of each image type (legend), and perfect correspondence (black dotted line) are shown.

To confirm that Viridot could reliably count plaques for other flavivirus strains and on different cell substrates, plaques were counted for neutralizing antibody titrations of additional viruses including DENV1-4 strains (n = 10 viruses) on C6/36 cells as well as a DENV2 and ZIKV strain on Vero cells. Concordance was >0.97 for the majority of strains, although concordance for some ZIKV and DENV1 titrations was lower (Fig 6A and S2 Table). The neutralization curves again show good agreement between manual and Viridot counted titrations, with an average ratio of PRNT_50_ titers of 0.94 [0.86–1.02] (Fig 6B and 6C and S3 Table), indicating slightly higher average neutralizing antibody titers with Viridot, although not significantly.

### Manual and Viridot plaque counts for well images taken with camera phones

**Fig 7:**
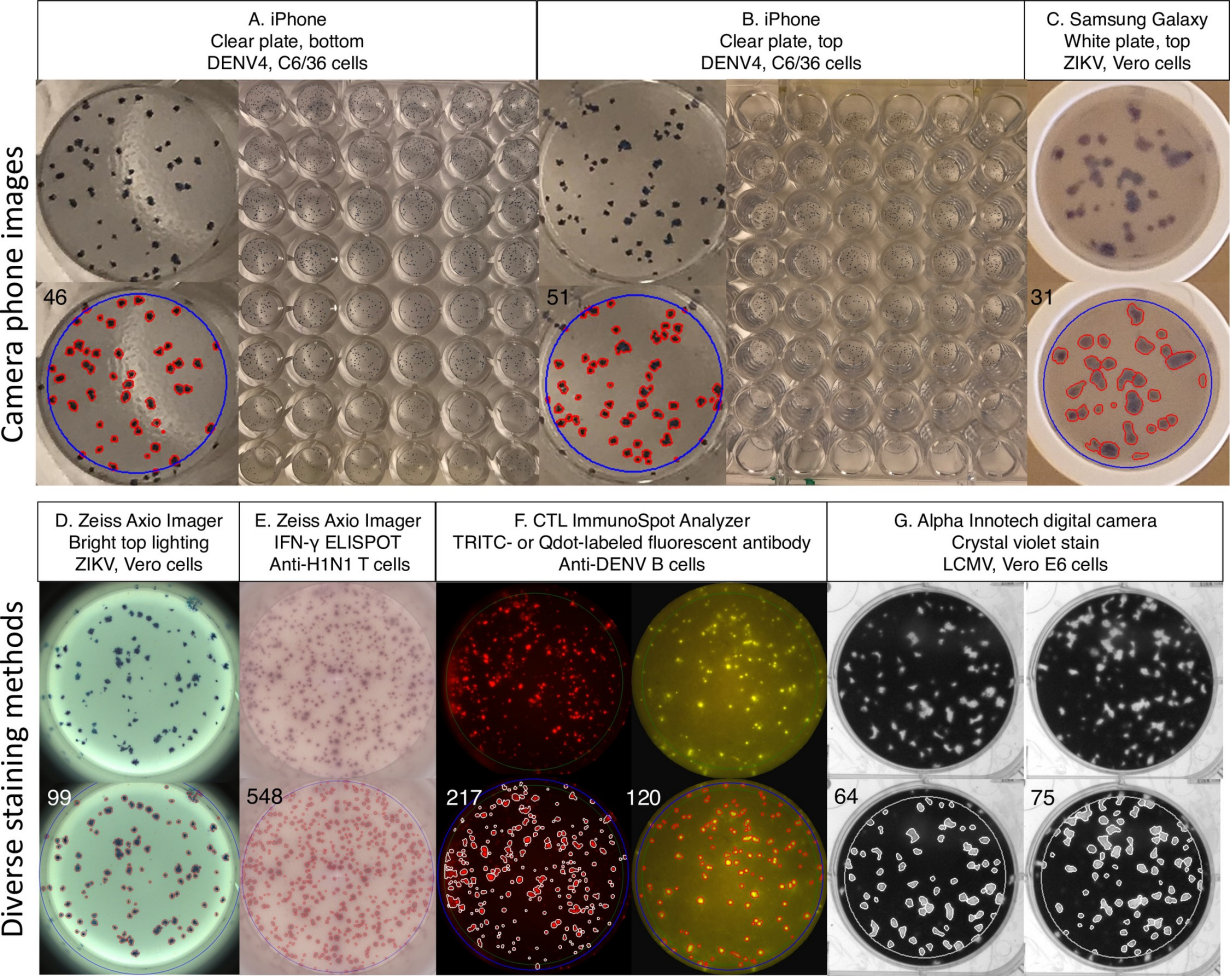
Viridot counting of camera phone well images and spots acquired using other staining methods. **(A and B)** Images of DENV4 plaques on C6/36 cells (peroxidase-labeled antibody and TrueBlue stain) acquired using the iPhone 7 plus of the bottom and top of the plate. **(C)** Images of ZIKV plaques on Vero cells on white plates, with images acquired using the Samsung Galaxy S7. **(D)** Images of ZIKV plaques on Vero cells acquired using the Zeiss Axio Imager.M1 microscope, using bright top lighting. **(E)** IFN-γ ELISPOT of the T-cell response to Influenza A nucleoprotein peptide 147–155, imaged with the Zeiss Axio Cam Imager.M2 scope with the AxioCam MRc camera, 5x objective lens. **(F)** Total (left) and DENV4-specific IgG^+^ memory B cells (right). Spots were detected with TRITC-labeled IgG-specific antibody or Qdot-conjugated fluorescent antibody using an ELISPOT based Fluorospot, with images acquired using the ImmunoSpot Analyzer. **(G)** LCMV plaques on Vero E6 cells resolved using crystal violet and imaged using the Alpha Innotech digital camera with the Computar H6Z0812M motorized zoom lens.

To evaluate whether Viridot could be used as part of a lower-cost image acquisition platform, we used camera phones to image plates from the top (well-side) and bottom of 96 well plates (Fig 7A–7C). A single photo of the bottom of a 96 well plate allowed for reliable counting of 30 wells from a single image. Overall, we observed strong concordance between manual and Viridot counted plaques from camera phone images (ρ_c_ from 0.85–0.96, Fig 6D).

### Diverse well images can be analyzed with Viridot

Viridot is based on standard image processing tools and thus can be easily adapted to count images with other types of spots. Fig 7 shows well images acquired with bright lighting, from T and B cell ELISPOT assays, and from plaque assays stained using crystal violet (Fig 7D–7G). To use Viridot on such images, the user must adjust the light setting (color channel) in step 1 of the plaque counter parameter-setting window.

## Discussion

In this study, we present Viridot (Viridot package) for R, a free, open-source suite of virology tools with a user-interface in shiny designed for viral plaque counting as well as for neutralizing antibody titer estimation. Viridot was designed by and for laboratory-based researchers performing DENV and ZIKV neutralizing antibody titrations with the goal of improving the speed and consistency of the PRNT, a critical assay for measuring protective anti-flavivirus antibodies. We demonstrate that Viridot can count plaques on well images from 96-well plates at least as reliably as two analysts counting manually from the printed well image, suggesting that Viridot can be used to help researchers transition to using 96-well plates for the PRNT.

Although manual plaque counting is the gold-standard approach, to our knowledge, few studies describe the rules used for manual plaque counting or efforts to standardize counting methods between analysts [33]. This may introduce additional variability into neutralizing antibody titer quantification beyond factors already identified [34,35]. We show that applying consistent rules for manual plaque counting both within and between analysts can improve consistency of plaque counts. An advantage of the Viridot plaque counter is that it allows users to develop and implement exact plaque-counting rules and apply those rules consistently across wells and plates, reducing the risk of operator bias and variation amongst analysts even in the same laboratory. On average, manual and Viridot counted plaques and PRNT_50_ titers were not significantly different. However, for certain titrations, including for certain viruses, Viridot did significantly deviate from manual counts, suggesting that inaccurate counting can occur. Undercounting mostly occurs for wells with large, irregularly shaped clusters of plaques that Viridot counts as a single plaque, while overcounting is most common in wells with pale plaques that are close in color intensity to background. Adjustment of parameter settings can help but may still be imperfect. Users should check Viridot counts of well images against their rules for manual counting to ensure the program is optimized to reflect user judgment about what constitutes a plaque.

Viridot provides some advantages over other existing tools. CTL Analyzer software and ImageJ require more extensive parameter optimization (clicking through menu-bars to identify all modifiable parameters) for each plaque phenotype, making them efficient programs for protocols using a few prototype strains but inconvenient for working with large numbers of diverse viral strains with distinct plaque phenotypes. Viridot has parameters readily accessible and organized in a single scroll bar, making optimization easier, and can be used for analysis of a diverse array of plaque phenotypes as well as for other immunostained spots. Further, the source code for the CTL ImmunoSpot Analyzer software is proprietary, and ImageJ is written in JavaScript, a language with which many laboratory-based researchers may not be familiar. Viridot is open-source and written in R, a statistical language used widely by biologists, potentially making it easier for researchers to modify the program for their own means. Further, while commercial counters have high-quality hardware for acquiring images of each well, they are expensive. We show that Viridot can analyze camera phone images, making it potentially useful in low-resource settings as part of an inexpensive plaque counting pipeline. With further development and standardization, Viridot may be useful in settings that involve stringent requirements such as the assay validation required for clinical trials or hospital settings.

Viridot has some limitations. The Viridot plaque counter was not fully optimized for all light and cellular conditions, and thus may perform better under some conditions than others. Unlike the ImmunoSpot Analyzers, Viridot does not have an interactive interface for quality control or manually removing incorrectly counted plaques. Viridot is not a commercial product and thus does not have a warranty.

Overall, Viridot was developed as a tool for researchers measuring neutralizing antibody responses to viruses using immunostain-based plaque reduction assays. Viridot has multiple advantages over manual plaque counting as well as other automated programs, enabling rapid counting of diverse viral strains on 96-well plates as well as a framework for formalizing plaque-counting rules to improve the consistency across experiments, analysts, and laboratories.

## Supporting information

S1 Fig The effect of each image-processing step in the Viridot plaque counting pipeline is shown from the original raw well image to the counted well image. The user can visualize each step using the "Show what is done at each step" option in the interface. Step 1: select light setting. Step 2: blur image (extra option to remove strings/fibers in image at this stage). Step 3: cut well edges and insert value for pixels outside well. Step 4: apply contrast to image based on background and plaque intensity. Step 5: select difference in pixel value to distinguish plaque from background and size (in pixels) of the window for applying the thresholding algorithm to the image. Step 6: dilate plaques to ensure they are counted as single objects. Step 7: cut overlapping plaques so they are counted separately. Step 8: define the minimum and maximum pixel size to count as a plaque. Details are provided in the *Step-by-step guide to optimizing plaque counter parameter settings* section of the Viridot manual (S1 Supporting File).
(PDF)

S2 Fig Comparison of manual plaque counts among analysts. Manual plaque counting by four separate analysts for **(A)** a well of uniform, evenly colored plaques or **(B)** a well of plaques with non-uniform size and intensity.
(PDF)

S1 Table Plate, camera, and color settings used for taking images of plaques using a CTL Immunospot Analyzer, corresponding to the Costar 96 well template built into the software. (DOCX)

S2 Table Manual and Viridot plaque count comparison as shown in Figs 5 and 6. (DOCX)

S3 Table Neutralizing antibody titers (PRNT_50_) estimated with manual and Viridot plaque counting (Data correspond to Fig 6). (DOCX)

S1 Supporting File The Viridot manual. (PDF)

S2 Supporting File PRNT and image acquisition protocols. (PDF)

S1 Supporting Data A .zip file that includes plaque count data used for Figs 5 and 6. (ZIP)
