# Supplementary figures and images for: Viridot: An automated virus plaque (immunofocus) counter for the measurement of serological neutralizing responses with application to dengue virus
Source: PLoS Negl Trop Dis. 2018 Oct 24;12(10):e0006862. doi: 10.1371/journal.pntd.0006862 (PMC6226209; doi:10.1371/journal.pntd.0006862)

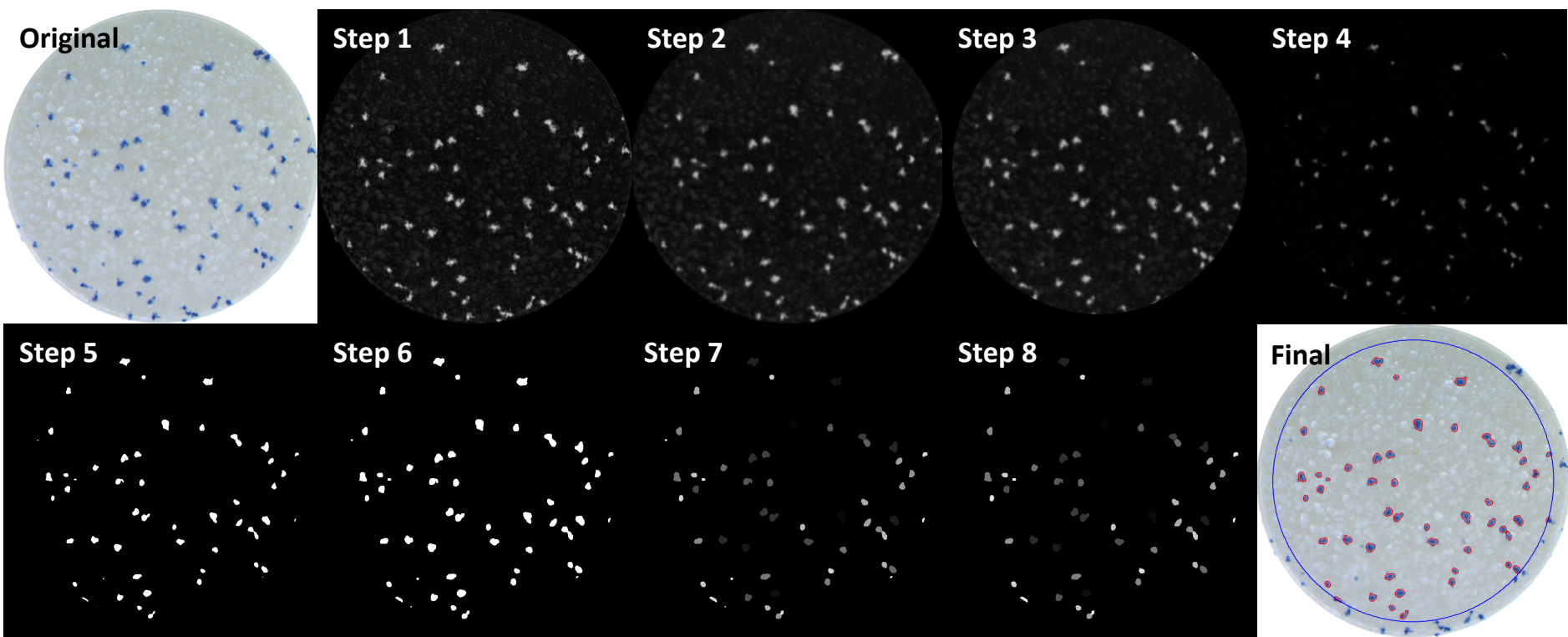

Figure S1

Supplement: S1 Fig — The user can visualize each step using the "Show what is done at each step" option in the interface. Step 1: select light setting. Step 2: blur image (extra option to remove strings/fibers in image at this stage). Step 3: cut well edges and insert value for pixels outside well. Step 4: apply contrast to image based on background and plaque intensity. Step 5: select difference in pixel value to distinguish plaque from background and size (in pixels) of the window for applying the thresholding algorithm to the image. Step 6: dilate plaques to ensure they are counted as single objects. Step 7: cut overlapping plaques so they are counted separately. Step 8: define the minimum and maximum pixel size to count as a plaque. Details are provided in the Step-by-step guide to optimizing plaque counter parameter settings section of the Viridot manual (S1 Supporting File). (PDF) [file pntd.0006862.s001.pdf]

A

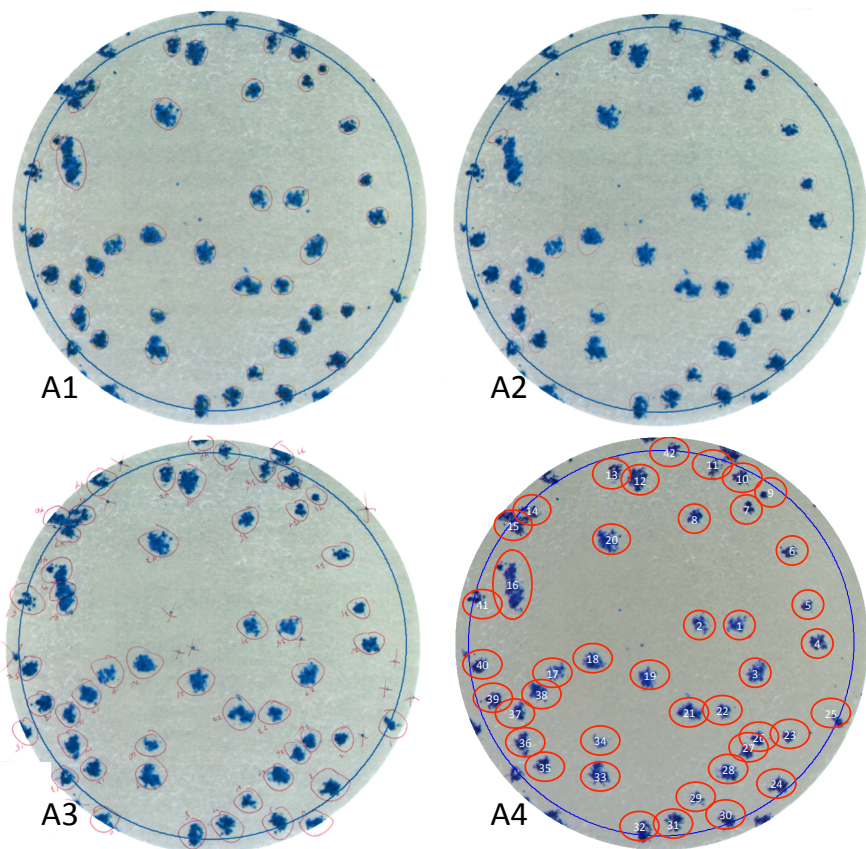

B

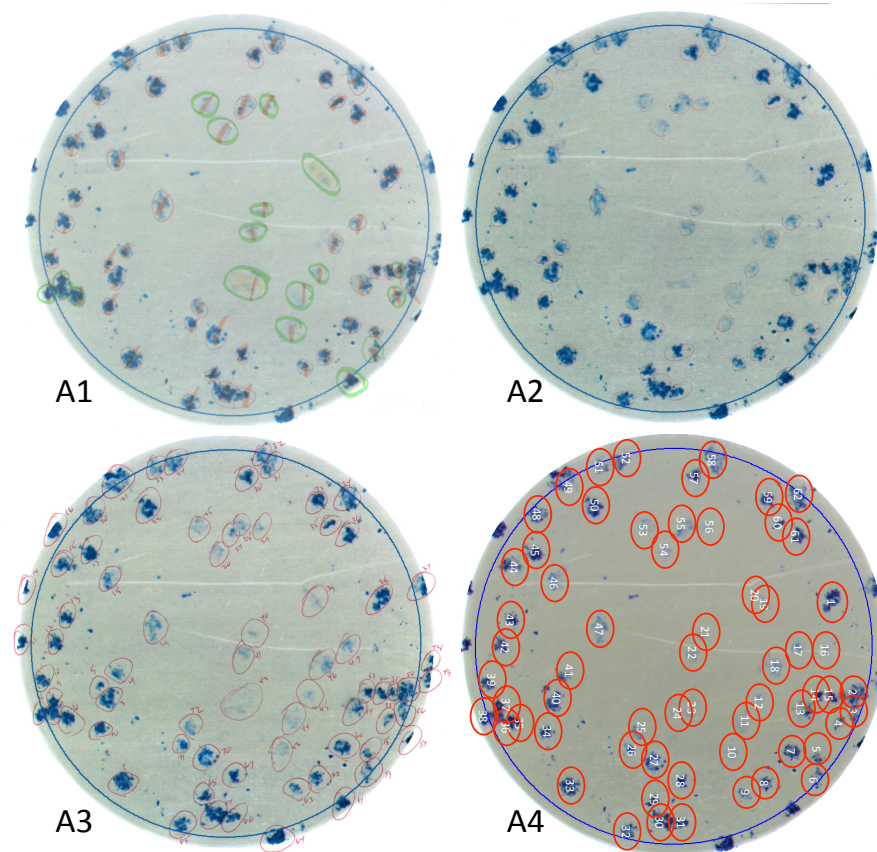

Figure S2

Supplement: S2 Fig — Manual plaque counting by four separate analysts for (A) a well of uniform, evenly colored plaques or (B) a well of plaques with non-uniform size and intensity. (PDF) [file pntd.0006862.s002.pdf]
