# Supplementary material for: Viridot: An automated virus plaque (immunofocus) counter for the measurement of serological neutralizing responses with application to dengue virus
Source: PLoS Negl Trop Dis. 2018 Oct 24;12(10):e0006862. doi: 10.1371/journal.pntd.0006862 (PMC6226209; doi:10.1371/journal.pntd.0006862)
Supplement: S1 Table — (DOCX) [file pntd.0006862.s003.docx]

| **Optional settings** | **Setting used** |
| --- | --- |
| **Filter changer** |  |
| Selected filter | 0 |
|  |  |
| **Plate profile details** |  |
| Plate profile name | Costar 96 Well |
| Format | 12 columns, 8 rows |
| Material: plate color | Clear |
| Material: bottom | Clear |
| Well diameter (mm) | 6.2 (well surface area=30.191) |
| Plate orientation | ROI radius (pixels) 383 |
| Light sources | Top |
|  |  |
| **Camera settings** |  |
| Camera profile | Normal resolution |
| Exposure setting | 0.0062 Units (Min: 0.0001, Max: 2) |
| Int. target % | 0 |
| Factor | 0 |
| Offset | 0.000 |
| AutoExp. limit | 2.000 |
|  |  |
| **Color settings** |  |
| Gain | 0.00 |
| Red | 1.33 |
| Green | 1.00 |
| Blue | 1.03 |
| Gamma | 0.90 |
| Saturation | 150 |
| Degrees | 6500 |
