# Supplementary material for: Viridot: An automated virus plaque (immunofocus) counter for the measurement of serological neutralizing responses with application to dengue virus
Source: PLoS Negl Trop Dis. 2018 Oct 24;12(10):e0006862. doi: 10.1371/journal.pntd.0006862 (PMC6226209; doi:10.1371/journal.pntd.0006862)
Supplement: S2 Table — (DOCX) [file pntd.0006862.s004.docx]

| *Titration name* | *Concordance ρ_c_ (95% CI)* | *Plaque count ratio (95% CI)* |
| --- | --- | --- |
| DENV3 T1, A1 | 1 | 1 |
| DENV3 T2, A1 | 1.00 [1.00-1.00] | 1.05 [0.94-1.17] |
| DENV3 T3, A1 | 0.99 [0.99-1.00] | 1.02 [0.99-1.07] |
| DENV3 T4, A1 | 0.99 [0.98-1.00] | 0.97 [0.93-1.02] |
| DENV3 T5, A1 | 1.00 [1.00-1.00] | 1.00 [1.00-1.00] |
| DENV3 T6, A1 | 0.99 [0.97-1.00] | 1.01 [0.98-1.04] |
|  |  |  |
| DENV3 T1, A2 | 1.00 [1.00-1.00] | 1.00 [0.99-1.01] |
| DENV3 T2, A2 | 1.00 [1.00-1.00] | 1.05 [0.95-1.17] |
| DENV3 T3, A2 | 1.00 [1.00-1.00] | 1.01 [0.99-1.02] |
| DENV3 T4, A2 | 1.00 [0.99-1.00] | 1.04 [0.96-1.13] |
| DENV3 T5, A2 | 0.99 [0.99-1.00] | 1.04 [1.01-1.07] |
| DENV3 T6, A2 | 0.98 [0.93-0.99] | 1.06 [1.00-1.13] |
|  |  |  |
| DENV1-a | 0.88 [0.78-0.93] | 0.91 [0.89-0.92] |
| DENV1-b | 1 | 1 |
| DENV1-c | 0.98 [0.96-0.99] | 1.07 [1.03-1.12] |
| DENV2-a | 0.99 [0.98-1.00] | 1.00 [0.98-1.02] |
| DENV2-b | 1.00 [1.00-1.00] | 1.00 [0.99-1.00] |
| DENV2-c | 0.98 [0.96-0.99] | 0.93 [0.89-0.97] |
| DENV3-a | 1.00 [0.99-1.00] | 0.99 [0.98-1.01] |
| DENV3-b | 0.99 [0.99-1.00] | 0.98 [0.95-1.02] |
| DENV4-a | 1.00 [1.00-1.00] | 0.99 [0.98-1.00] |
| DENV4-b | 0.99 [0.98-1.00] | 0.98 [0.95-1.00] |
| Zika-a | 0.92 [0.86-0.95] | 1.00 [0.94-1.07] |
| Zika-b | 0.99 [0.99-1.00] | 1.02 [0.99-1.05] |
| Zika-c | 0.74 [0.54-0.86] | 0.91 [0.85-0.97] |
| DENV2-vero | 0.97 [0.96-0.98] | 0.94 [0.92-0.96] |
