# Supplementary material for: Viridot: An automated virus plaque (immunofocus) counter for the measurement of serological neutralizing responses with application to dengue virus
Source: PLoS Negl Trop Dis. 2018 Oct 24;12(10):e0006862. doi: 10.1371/journal.pntd.0006862 (PMC6226209; doi:10.1371/journal.pntd.0006862)
Supplement: S3 Table — (DOCX) [file pntd.0006862.s005.docx]

|  | *Manual* | *Viridot* |
| --- | --- | --- |
|  | *1/serum dilution* | *1/serum dilution* |
| DENV1-a | <10 [1-13] | <10 [1-12] |
| DENV1-b | 26 [10-60] | 26 [10-60] |
| DENV1-c | 115 [36-297] | 129 [41-334] |
| DENV2-a | 37 [22-54] | 36 [23-50] |
| DENV2-b | 757 [481-1202] | 842 [538-1330] |
| DENV2-c | 558 [405-734] | 614 [468-785] |
| DENV3-a | 13 [2-31] | 11 [2-27] |
| DENV3-b | 423 [282-627] | 399 [279-566] |
| DENV4-a | 93 [32-272] | 110 [46-305] |
| DENV4-b | 432 [227-811] | 441 [227-827] |
|  |  |  |
|  | *MAb concentration (ng/mL)* | *MAb concentration (ng/mL)* |
| Zika-b | 155 [364-53] | 206 [585-82] |
| Zika-c | 139 [341-54] | 162 [392-66] |
