# Supplementary material for: Viridot: An automated virus plaque (immunofocus) counter for the measurement of serological neutralizing responses with application to dengue virus
Source: PLoS Negl Trop Dis. 2018 Oct 24;12(10):e0006862. doi: 10.1371/journal.pntd.0006862 (PMC6226209; doi:10.1371/journal.pntd.0006862)
Supplement: S1 Supporting File — (PDF) [file pntd.0006862.s006.pdf]

Viridot Manual

(This manual corresponds to the version of Viridot available on GitHub as of October 13, 2018. For more recent versions, please visit: https://github.com/leahkatzelnick/Viridot.)

Table of Contents

1. VIRIDOT INSTALLATION INSTRUCTIONS 4

1.1. R AND RSTUDIO INSTALLATION 4
1.2. GETTING VIRIDOT FROM GITHUB 4
1.3. VIRIDOT MAC INSTALLATION INSTRUCTIONS 4
1.4. VIRIDOT WINDOWS INSTALLATION INSTRUCTIONS 6

2. QUICK-START: WORKING THROUGH EXAMPLES 7

2.1. PLAQUE COUNTING EXAMPLE 7
2.1. NEUTRALIZATION TITER ESTIMATION EXAMPLE 8

3. PLAQUE COUNTER TAB 10

3.1. SELECT IMAGES TO ANALYZE FOR WHEN OPTIMIZING PLAQUE COUNTING PARAMETERS OR WHEN CONDUCTING YOUR FINAL PLAQUE COUNTS (CENTER PANEL) 10
(1) SELECT THE DIRECTORY THAT CONTAINS YOUR PLATE FOLDERS 10
(2) SELECT THE PLATE FOLDERS THAT CONTAIN WELL IMAGES TO COUNT 11
(3) SELECT THE WELL IMAGES TO COUNT 11
SUBMIT 12
WARNING MESSAGES APPEAR HERE: 12
3.2. SAVING ANALYZED IMAGES AND PLAQUE COUNTS (RIGHT PANEL) 12
ARE THESE IMAGES PART OF A PLATE? 13
SAVE PLAQUE COUNT TABLES? 13
PRINT OUTLINES OF PLAQUES ON ALL IMAGES? 13
OUTPUT SPREADSHEETS OF PLAQUE SIZES? 14
UNIQUE NAME TO INCLUDE SAVING PLAQUE TABLES AND/OR IMAGES 14
DIRECTORY FOR SAVING PLAQUE TABLES/IMAGES 14
3.3. PLAQUE COUNTER PARAMETER SETTINGS (LEFT PANEL) 14
LOAD PREVIOUS PLAQUE COUNTER PARAMETER SETTINGS 14
SAVE CURRENT PLAQUE COUNTER PARAMETER SETTINGS 15
3.4. STEP-BY-STEP GUIDE TO OPTIMIZING PLAQUE COUNTER PARAMETER SETTINGS 15
SHOW WHAT IS DONE AT EACH STEP 15
STEP 1. SELECT LIGHT SETTING 17
STEP 2. BLUR IMAGE 18
EXTRA OPTION: REMOVE STRINGS/FIBERS IN IMAGE 19
STEP 3.1. CUT WELL EDGES 19
STEP 3.2. INSERT VALUE FOR PIXELS OUTSIDE WELL 20
STEP 4.1. APPLY CONTRAST TO IMAGE BASED ON BACKGROUND INTENSITY 20
STEP 4.2. APPLY CONTRAST TO IMAGE BASED ON PLAQUE INTENSITY 21
STEP 5.1. SELECT DIFFERENCE IN PIXEL VALUE TO DISTINGUISH PLAQUE FROM BACKGROUND 21

Step 5.2. Select size (in pixels) of the window for applying the thresholding algorithm to the image 22
Step 6. Dilate your plaques to ensure they are counted as single plaques 22
Step 7. Cut overlapping plaques so they are counted separately 22
Step 8.1. Define the minimum pixel size to count as a plaque 23
Step 8.2. Define the maximum pixel size to count as a plaque 23

4. NEUTRALIZATION TITER ESTIMATION TAB 23

4.1. Load files and plot data (CENTER PANEL, FIRST TAB) 23
Select the directory that contains your plaque count data 24
Select the table/tables of plaque counts 24
Select template 24
Estimate neut. titers 25
Warning messages appear here: 25
4.2. Template and tables (CENTER PANEL, SECOND TAB) 25
4.3. Saving neutralization titers (RIGHT PANEL) 25
Save neutralization curves and data? 25
Select the directory for saving neutralization data 25
4.4. Provide names for samples and experiment (LEFT PANEL) 25
Sample names 25
Virus names 26
Experiment name 26
Run only a subset of samples 27
4.5. Provide information on dilution series and neutralization titer estimation (LEFT PANEL) 27
Starting serum/antibody dilution 27
Dilution series (e.g. 2=2-fold, 3=3-fold, 4=4-fold) 27
Final serum/antibody dilution 27
Number of decimal places to print for titers/CI/slope 28
X-axis label 28
Show serum dilutions selected 28
Lower titer limit 28
Upper titer limit 29
LID Statistical Web Tools PRNT generator method? 29
Allow resistant fraction? 29
Percent plaque reduction 29
Number of plaques in control wells 30
4.6. Choices for plotting neutralization curves 30
Color of confidence interval 30
Color of regression line 30
Plot duplicate values 30

5. OTHER VIROLOGY TOOLS TAB 31

5.1. Estimate dilution needed to achieve a certain number of plaques per well for neutralization experiments 31
Titer of initial virus stock (X, as 10^X) 31
Target number of plaques per well in experiment 31
Final volume of diluted virus required for full experiment (in mL) 31
Volume of virus added to wells of cells in experiment (in mL) (* Virus only, not total serum+virus

MIXTURE) ... 31
ESTIMATE VIRUS DILUTION ... 31
5.2. ESTIMATE VIRUS TITER FROM A VIRUS TITRATION ... 32
STARTING DILUTION OF VIRUS TITRATION (FIRST ROW ON PLATE, E.G. 1/10) ... 32
WHAT DILUTION SERIES DID YOU USE FOR YOUR VIRUS TITRATION (E.G. 10-FOLD) ... 32
WHICH ROW ON PLATE (WHICH DILUTION NUMBER) ARE THE PLAQUE COUNTS INPUT HERE FROM? ... 32
VOLUME TRANSFERRED TO WELLS OF CELLS IN VIRUS TITRATION (IN ML) ... 32
NUMBER OF PLAQUES (CAN INPUT MULTIPLE NUMBERS SEPARATED BY A COMMA) PER WELL COUNTED ... 32
ESTIMATE VIRUS TITER ... 32

6. IMAGE FORMATTING ... 33

DIRECTORY OF IMAGES TO BE TRIMMED ... 33
SELECT THE WELL IMAGES TO TRIM ... 33
WHAT DO YOU WANT TO DO? ... 33
DIRECTORY FOR SAVING TRIMMED IMAGES ... 33
PRINT OUT INDIVIDUAL SQUARE WELLS FROM SINGLE LARGE IMAGE? ... 33
HOW MANY PIXELS PER WELL? ... 33
HOW FAR FROM THE LEFT OF THE IMAGE SHOULD THE GRID START? ... 33
HOW FAR FROM THE TOP OF THE IMAGE SHOULD THE GRID START? ... 34
TRIM! ... 34

1. Viridot installation instructions

1.1. R and RStudio installation

R [18] and RStudio (RStudio makes it easier to learn R) can be downloaded from the following website: https://www.rstudio.com/products/rstudio/download/#download

Make sure to select the version of R and RStudio that correspond to your operating system. Viridot was written to work with versions up to R 3.4.1.

1.2. Getting Viridot from GitHub

You can download Viridot from GitHub by visiting the following webpage: https://github.com/leahkatzelnick/Viridot. On the webpage, press the green button that says "clone or download". If you choose to clone Viridot, it will save a folder to your computer called Viridot. If you download the package as a .zip file, it will save a folder to your computer as a folder called Viridot-master or a .zip file called Viridot-master.zip. If it is a .zip file, double click on it to unzip it. A folder with the same name as the .zip file should now appear. **Please rename the Viridot-master folder Viridot.** The Viridot folder can be saved anywhere on your computer.

Note: Mac created .zip files unzip differently in Windows, so you will need to duplicate the final path folder. Mac zipped files unzip into a folder with the folder you want and a _MACOSX folder. Another option is to delete the MACOSX folder and copy everything into the first directory.

1.3. Viridot Mac installation instructions

Viridot can be installed on a Mac by following these instructions:

Open RStudio and type into the console:

```
install.packages("~/Downloads/Viridot", repos=NULL,
type="source")
```

Make sure to replace the "~/Downloads/" section with wherever you have saved the Viridot package on your computer. For instance, if you have saved Viridot to your Desktop, you tell R to look in the home directory (~) in the folder (/) called Desktop, and then in the Desktop folder (/), there is a folder called Viridot:

```
install.packages("~/Desktop/Viridot", repos=NULL, type="source")
```

You can also have R write the path for you by dragging in the file to the console, holding it over the space where you want the path written, and when you see a green “+” sign where your cursor is, you can let go of the file. Make sure your path is inside quotation marks.

If 'done Viridot' is printed in your console after running the above command, Viridot installed! Viridot should now be loaded into R and/or RStudio. No need to repeat the install.packages() step next time you open RStudio again to use Viridot—you only need to install the program once.

Each time you open RStudio, you will need to do the following to load and open Viridot:

```
require(Viridot)
launchViridot()
```

The first time you type launchViridot(), the various packages Viridot uses, including shiny [19], shinyFiles [21], EBImage [20], gtools [22], and drc [23], should be automatically installed. R will ask if you want to install these and other dependencies for Viridot: make sure you type 'y' to indicate yes.

To start from the "examples" directory available in Viridot, run the following:

```
require(Viridot)
volumes <- setVolumes(location="examples")
launchViridot()
```

To easily navigate to directories where *your* plate folders (containing well images for a plate) or data files are stored, you will want to specify a starting directory other than the example directory. To do this, you can change the *volumes* object, for instance by starting in the Documents folder:

```
require(Viridot)
volumes  <-  setVolumes(location="~/Documents", name.volumes="my-
data")
launchViridot()
```

You can change "my-data" to a name of your choice, so long as you keep the quotation marks around the name. (Make sure they are quotation marks as shown in this document, not smart quotation marks).

You can also tell Viridot to start in your home directory. On a Mac, this can be done by running the following commands:

```
require(Viridot)
volumes <- setVolumes(location="homeDirectoryForMac")
```

```
launchViridot()
```

1.4. Viridot Windows installation instructions

Viridot can be installed in Windows by following these instructions:

Open RStudio, and in the console, run the following command:

```
install.packages("PATH/Viridot", repos=NULL, type="source")
```

Where PATH corresponds to where the unzipped folder is located on your computer. For example, if the Viridot folder is saved in *C:/*, copy the following command into the R console:

```
install.packages("C:/Viridot", repos=NULL, type="source")
```

You should now have installed Viridot. To load Viridot in R, open R or RStudio and type:

```
require(Viridot)
```

Because Viridot is automatically configured for Mac, you need to change the object *volumes* so that Windows knows where your data, images, and analysis files are located. For example, if you have saved your data for analysis in a drive called C:/, you would type the following into your console:

```
volumes <- setVolumes(location="C:/")
names(volumes) <- "C"
```

You can also run the following to save to *C:/* on Windows:

```
volumes <- setVolumes(location="homeDirectoryForWindows")
```

Folders inside a drive work fine, too. For example, if you have a folder within the C drive called Experiments you could write:

```
volumes <-setVolumes(location="C:/Experiments", name.volumes=("C
- PLAQUE COUNTER")
```

The names.volumes selection can be whatever you want it displayed as in R, so instead of “R – PLAQUE COUNTER” it could be called “My data”.

Note: When using the drive itself, you need the slash, “C:/” or “R:/”. However, when using a folder, you need to leave the last slash off or it won’t work “C:/Folder”, NOT “C:/Folder/”.

You may now launch Viridot by typing this command into the RStudio console:

```
launch.viridot()
```

The first time you type launchViridot(), the various packages Viridot uses, including shiny [19], shinyFiles [21], EBImage [20], gtools [22], and drc [23], should be automatically installed. R will ask if you want to install these and other dependencies for Viridot: make sure you type 'y' to indicate yes.

2. Quick-start: working through examples

2.1. Plaque counting example

Open RStudio. We assume you have already installed Viridot (see previous section). In the R console, type the following three commands to launch Viridot (this assumes you have a Mac, please see the above section for Windows instructions):

```
require(Viridot)
volumes <- setVolumes(location="examples")
launchViridot()
```

In the center of the screen is the ***Select images to analyze*** panel. Press the ***Select the directory that contains your plate folders*** button (in the top center of the screen). Click on *Example_directory_of_plates* so that it is highlighted and press ***Select***. The box that previously said "character(0)" should now print out the path to the example directory of plate folders.

Now click on the box below the title ***Select the plate folders that contain well images to count***. Press *Example_plate_1*. *Example_plate_1* should now appear in the box. Then click on any blank space outside the box to finalize the selection.

Now click on the box below the title ***Select the well images to count***. Choose the file named *Example_well_1.tif. Example_well_1.tif* should now appear in the box. Click on any blank space outside the box to finalize the selection. Overall, the set up should now look like this with your path in the first option:

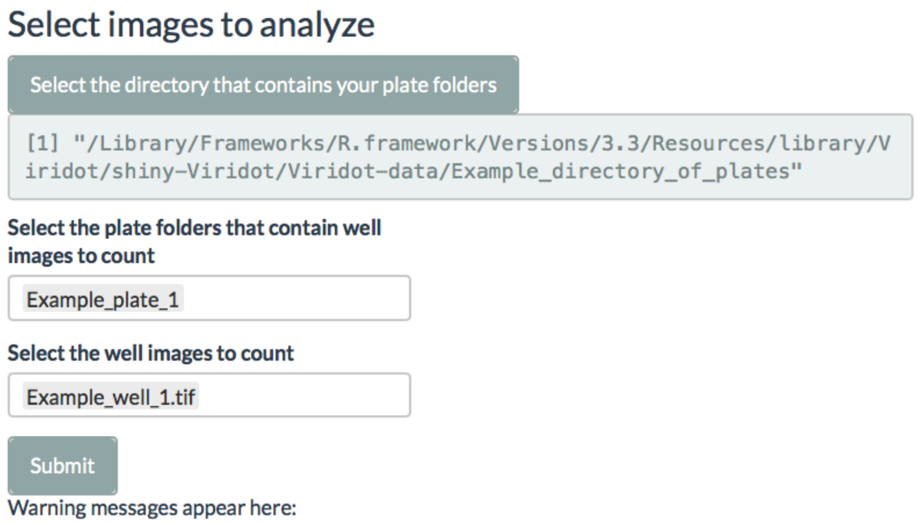

Now press the ***Submit***. The counted image should now appear below the ***Well outline color*** box (at the bottom of your screen). Two new boxes appear: one with the title ***Well name***, which contains the name of the image file being shown, and one with the title ***Number of plaques counted in this well***, which contains the number of plaques counted for that well.

The plaque counter parameter settings are available in the scroll bar on the left of the screen. Try modifying these values and checking the resulting effect on plaque counts by pressing ***Submit***.

2.1. Neutralization titer estimation example

Open R Studio. We assume you have already installed Viridot (see previous section) In the R console, type the following two commands to launch Viridot (again we assume you are using a Mac):

```
require(Viridot)
volumes <- setVolumes(location="examples")
launchViridot()
```

At the top of the screen, select the ***Neutralization titer estimation*** tab.

To load a plate to count, press the button ***Select the directory that contains your plaque count data***. Within that window, click on the folder

Example_plates_with_plaques_counted and press ***Select***.

Now click on the box below the title ***Select the table/tables of plaque counts***. Choose the file named *plaque-count-table-96-wells.xls.* It should now appear in the box. Click on any blank space outside the box to finalize the selection.

Now, click on the **Template and *tables*** tab. The table you just loaded will now appear.

Click back over to the ***Load files and plot data*** tab. Press the ***Estimate neut. titers*** button. The neutralization curves should now appear. Parameter settings for estimating and plotting the neutralization curves are available in the scroll bar on the left of the screen.

To see the plate template used for knowing where the neutralization titrations and controls are on your plate, click on the ***Template and tables*** tab again. Under the title ***Plate template for analysis***, you will now see the default plate template (corresponds to *template_96_well_plate_default.csv* in the folder in the examples directory entitled *Example_templates_of_plates_for_neutralization_titer_estimation*). The default template (shown here) assumes that you have imported a 96 well plate, have four different samples (S1, S2, S3, and S4) each with 12 dilutions (D1, D2, D3, D4, D5, D6, D7, D8, D9, D10, D11, D12) and each titration is done in duplicate (there are two wells on each plate entitled S1-D1, etc.):

Plate template for analysis

	X1	X2	X3	X4	X5	X6	X7	X8	X9	X10	X11	X12
A	S4-D1	S4-D2	S4-D3	S4-D4	S4-D5	S4-D6	S4-D7	S4-D8	S4-D9	S4-D10	S4-D11	S4-D12
B	S4-D1	S4-D2	S4-D3	S4-D4	S4-D5	S4-D6	S4-D7	S4-D8	S4-D9	S4-D10	S4-D11	S4-D12
C	S3-D1	S3-D2	S3-D3	S3-D4	S3-D5	S3-D6	S3-D7	S3-D8	S3-D9	S3-D10	S3-D11	S3-D12
D	S3-D1	S3-D2	S3-D3	S3-D4	S3-D5	S3-D6	S3-D7	S3-D8	S3-D9	S3-D10	S3-D11	S3-D12
E	S2-D1	S2-D2	S2-D3	S2-D4	S2-D5	S2-D6	S2-D7	S2-D8	S2-D9	S2-D10	S2-D11	S2-D12
F	S2-D1	S2-D2	S2-D3	S2-D4	S2-D5	S2-D6	S2-D7	S2-D8	S2-D9	S2-D10	S2-D11	S2-D12
G	S1-D1	S1-D2	S1-D3	S1-D4	S1-D5	S1-D6	S1-D7	S1-D8	S1-D9	S1-D10	S1-D11	S1-D12
H	S1-D1	S1-D2	S1-D3	S1-D4	S1-D5	S1-D6	S1-D7	S1-D8	S1-D9	S1-D10	S1-D11	S1-D12

Lets assume that actually, rows A and B on the plate contain controls. To change the template, click the tab ***Load files and plot data*** and press the ***Select template*** button. Double click on the *Example_templates_of_plates_for_neutralization_titer_estimation*

folder and within that, select *template_96_well_plate_with_controls.csv*. Press the **Select** button. Click back over to the ***Template and tables*** tab, and now the format for *template_96_well_plate_with_controls.csv* should appear. The template included wells labeled C1, C2, and C3. These indicate to the program that these wells contain three distinct controls.

Click the tab ***Load files and plot data*** and press the ***Estimate neut. titers*** button.

You will receive an error, saying: "The number of serum names given doesn't match the number of samples in your template. Please correct this." On the scroll bar on the left, under the section ***Provide names for samples and experiment***, and the title ***Sample names***, remove the final serum name, SR-4. Make sure there are still commas between the names (e.g. *SR-1,SR-2,SR*-3). Any spaces between commas will be assumed to be a part of the name. Now press the ***Estimate neut. titers*** button again. The neutralization curves with the updated names will now appear. Above the neutralization curves, you will see a table that indicates the summary statistics for the three controls 1, 2, and 3.

3. Plaque Counter tab

3.1. Select images to analyze for when optimizing plaque counting parameters or when conducting your final plaque counts (CENTER PANEL)

(1) Select the directory that contains your plate folders

Click this button to select the directory that contains the plate folders you want to analyze. A small window will open. You will click on the directories listed in the left window to find the directory containing your plate folders (the window on the right only tells you what is in that directory). Once you have selected the directory you want, press *Select*.

The structure you want for this selection and the next two selections— (1) directory of plates, (2) plate folders, and (3) wells images—should follow this scheme:

Scheme 1. Data directory structure.

- ***Directory that contains your plate folders***
  - Plate 1
    - …
  - Plate 2
    - Well 1
    - Well 2

- Well 3
    - …

- Plate 3
    - Well 1
    - Well 2
    - Well 3
    - …
- …

(2) Select the plate folders that contain well images to count

Once you have selected the directory that contains your plate folders, click on the box below the title ***Select the plate folders that contain well images to count***. In that box, you should see a list of the plate folders. (Actually, any file in the directory selected in ***(1)*** will be shown; you will have to know which are plate folders and which are other files.) If you are optimizing plaque-counting parameters, select one plate folder that contains the well images you plan to analyze. If you are conducting a final plaque count for your experiments, select one or more plate folders that contain the well images you plan to analyze.

Notes:

- You must select a FOLDER, not a file. If you select a file, and then select *all* under the setting ***Select the well images to count*** you will get the error: " You have either selected a file (rather than a folder) in the box under the title ***Select the plate folders that contain well images to count***, or the plate folder you selected has nothing in it".
- If you select multiple plates to analyze, only the image names for the first folder in the list will be shown in the box ***(3) Select the well images to count***.

(3) Select the well images to count

Once you have selected at least one plate folder containing well images of interest, click the box beneath the title ***Select the well images to count***, e.g. A1.tif. If you are optimizing plaque-counting parameters, select one well image file to count. If you are conducting a final plaque count for your experiments, select one or more well images to count. You can also select *all*, which will count plaques for all square image files found in the plate directories you have selected.

Notes:

- Images must be square (e.g. 700x700 pixels).
- Images must be in one of the following file formats: jpeg (or jpg), png, or tiff (or tif). .CTL files can be analyzed, as they are simply encrypted tiff files.
- Images smaller than ~300x300 pixels may not be effectively analyzed using the

saturation for ***Step 1. Select light setting***.

- If you try to read in a folder, text file, or other type of image file (e.g. pdf) instead of one of the accepted file formats, the program will give you the error "object 'image.to.display' not found".
- If you are counting more than one image, only the image and parameter settings for the LAST IMAGE IN THE QUEUE will be shown.

Once you have selected a well image to analyze (the well image cannot be specified in anything other than in the box ***Select the well images to count***) you can press the ***Submit*** button.

If you have selected only one well image to count, the well image will appear with the plaques that have been counted circled. The file name for the counted well image will appear in the box ***Well name*** and the plaque count is printed in the box ***Number of plaques counted in this well***. The well will be counted with whatever the plaque counting parameters are set to in the scroll bar on the left (see below). If you have NOT modified the parameter settings, the image will be analyzed with the default settings.

If you have selected more than one well image to count, only the last image will be shown in the window. Thus, you want to ensure that you are actually saving your data (see next section) if you are counting more than one image at a time.

Submit

By pressing submit, you initiate the plaque counting! If you have instructed the program to save data files or images, you must press ***Submit*** to actually save the data files and images.

Warning messages appear here:

Any error messages and warnings will appear here, for instance if you ask to count plaques in a text file rather than an image file, if you select an image file in anything other than the box "Select the well images to count", etc. (see notes above in section ***(3) Select the well images to count***).

3.2. Saving analyzed images and plaque counts (RIGHT PANEL)

Select where to save images of counted wells and plaque count tables with the ***Directory for saving plaque tables/images*** button. Once you select items here, go to the middle panel and press ***Submit*** again.

<u>Scheme 2.</u> Output data directory scheme

- Directory for saving plaque tables/images*
    - Plate 1
        - …

- Plate 2
  - Well 1
  - Well 2
  - Well 3
  - …
- Plate 3
  - Well 1
  - Well 2
  - Well 3
  - …

Notes:

- Your Output directory SHOULD NOT be the same as the ***Directory containing plate folders***. If you tell Viridot to use ***Directory containing plate folders*** as your ***Directory for saving plaque tables/images***, Viridot will insert the circled well images and plaque sizes into the directories containing your original data. It will overwrite your original data if you have not included a unique name in the box ***Unique name to include saving plaque tables and/or images***. Make sure to specify a different directory for ***Directory for saving plaque tables/images.***

Are these images part of a plate?

Choices include a *96-well plate* format, *24-well plate* format, or *Not a plate*. For a 96-well plate, the table will be organized such that the plaque count for the well image named *A1* will appear in the top left corner, *A12* in the top right corner, *H1* in the bottom left corner, and *H12* in the bottom right corner. If you have well images that are not part of a 96-well plate or a 24-well plate, or do not have well names that correspond to the wells on a plate (e.g. A1, B3, etc.) you can select *Not a plate*. The plaque count will be printed as a table with the well image name in the first column, and the corresponding plaque count in the second column.

Save plaque count tables?

You must tell Viridot that you want to make a data table of plaque counts. Select *Yes* to write the plaque counts table to the directory specified with the button ***Directory for saving plaque tables/images***. If you do not want to make a table of plaque counts at this time, select *No*.

Print outlines of plaques on all images?

For your records or as part of the optimization process, you may want to save images of the wells with the plaques circled. Select *Yes* to save circled well images for all the wells in which you are counting plaques. Select *No* if you do not want to save well images.

The circled well images will be saved into a folder with the same name as the plate folder name shown in the box (***2) Select the plate folders that contain well images to count.*** These plate folders will appear in the directory selected with the button ***Directory for saving plaque tables/images***.

Output spreadsheets of plaque sizes?

Viridot gives you the option of making plaque sizes for each well (select *Yes* for printing plaque sizes and *No* for not printing plaque sizes). Plaque sizes will correspond to the area of the circles in the circled well images. You should make sure that the circles correspond to the edges of the plaques by optimizing the plaque counting parameters to your particular plaque phenotype. The table of plaque sizes will be printed into the plate folders within the directory specified with the button ***Directory for saving plaque tables/images***. There will be a single spreadsheet of plaque sizes for each well.

Unique name to include saving plaque tables and/or images

Write in a unique name to use for saving your plate tables and/or counted well images (assuming you have selected to do this with the options above). The default name is *my-unique-name*. To add your own name, delete *my-unique-name* and enter a unique experiment name or number. This will help you TO AVOID OVERWRITING AN EXISTING PLAQUE-COUNT TABLE.

Directory for saving plaque tables/images

This button will open a window where you can select the folder where you would like to save the analyzed images and tables of plaque counts. After selecting the desired folder, press ***Select***. After you have selected the folder for saving tables/images, press the ***Submit*** button again and the counter will begin analyzing images and creating folders for each plate directory containing counted images and plaque sizes (if these options were selected above). Plaque count tables appear directly in the directory specified in ***Directory for saving plaque tables/images.***

3.3. Plaque counter parameter settings (LEFT PANEL)

Load previous plaque counter parameter settings

This option allows you to load previously saved plaque counter parameter settings. The instructions on how to save the values for parameters can be found below in the section ***Step-by-step guide to optimizing plaque counter parameter settings***.

Save current plaque counter parameter settings

This option allows you to save the values for each parameter. You will need to provide a name and a location for where the file is to be saved. In later experiments, these saved settings can be loaded and applied to a new set of images using the ***Load previous Plaque count parameter settings*** button.

3.4. Step-by-step guide to Optimizing plaque counter Parameter settings

Show what is done at each step

This tool allows you to look at the image during each step in the image-processing pipeline. The steps are additive, so the step 2 image includes the effects of plaque counting parameters specified in both step 1 and step 2, etc. The ***Show what is done at each step*** option allows you to understand how each parameter choice affects image processing and the final number of counted plaques. This is where you should go if you want to understand, for instance, whether you have chosen the correct light setting in Step 1, the correct amount of blur in Step 2, the right amount of contrast in Step 4, etc.

The following choices are available:

- Raw well image
- Step 1. Select light setting
- Step 2. Blur image
- Extra option: Remove strings/fibers in image
- Step 3. Cut well edges and insert value for pixels outside well
- Step 4. Apply contrast to image based on background and plaque intensity
- Step 5. Select difference in pixel value to distinguish plaque from background and size of the window for applying the thresholding algorithm to the image
- Step 6. Dilate your plaques to ensure they are counted as single plaques
- Step 7. Cut overlapping plaques so they are counted separately
- Step 8. Define the minimum and maximum pixel sizes to count as plaques
- Final counted plaques with well radius shown

Plaque counting with automatic parameter identification?

If you would like the program to try to automatically detect the best parameters for analyzing your image, click on the dropdown menu and select *Auto*. Some parameters are set to the default when you run *Auto*, unless changed by you (detailed below). If you would like to set all parameters yourself, select *User-specified*.

The *Auto* function of Viridot counter analyzes the image data for each well to identify parameters that best distinguish plaque from background. From each well image, the

darkness/size of the plaque, *P,* and the amount of background, *B*, are estimated (note: pixel values range from 0 to 1). *P* is estimated for each image as: (pixels with intensity >0.8)/(pixels with intensity >0.5). (Note: if there are no pixels >0.5, the denominator is set to 1 pixel). Dark, large plaques have high values of *P*, while pale, small plaques have low values of *P*. Parameter *P* is used to set the amount of Gaussian blur (***Step 2***), contrast (***Step 4.2***), and thresholding difference (***Step 5.1***). Parameter *B* is estimated as the peak in the histogram of pixel intensities for each image, excluding white pixels around the edge of the circular well. *B* is used to set the value for pixels outside the well (***Step 3.2***) and contrast (***Step 4.1***).

The other parameters are set to the default, but you can modify them at any time and the program will not automatically update them. If you load previous parameter settings, then run *Auto*, the parameters that do change (***Steps 2, 3.2, 4.1, 4.2, and 5.1***) will change (you will see the slider update to the setting used for the counted image) but the other parameters will stay at whatever you set them to before pressing ***Submit***.

Scheme 3. Automatic parameter identification mode.

Step 1. Select light setting
The program is designed for the saturation channel, but you can change it to some other light channel if you want.

Step 2. Blur image

	P	***Value***
Small, pale plaques	< 0.1144	2
Large, dark plaques	≥0.1144	3

Step 3.1. Cut well edges
Select any value (0-300).

Step 3.2. Insert value for pixels outside well

	B	***Value***
Super dark background	>0.6	Median background pixel value
Normal	≤ 0.6	Black (0)

Step 4.1. Apply contrast to image based on background intensity

	B	***Value***
Dark background	>0.2	0.5
	>0.15 & ≤ 0.2	0.75
	≥0.06 & ≤ 0.15	1
	0.05	1.5
	0.04	2

	0.03	2.5
Light background	≤0.02	3

Step 4.2. Apply contrast to image based on plaque intensity

	P	*Value*
Small, pale plaques	< 0.1144	2
Large, dark plaques	≥0.1144	3

Step 5.1. Select difference in pixel value to distinguish plaque from background

	Value
Default	0.08
If ***Step 2. Blur image*** Value ==2	0.12
If *P* <0.06	0.10

Step 5.2. Select size (in pixels) of the window for applying the thresholding algorithm to the image
Select any value (10-40).

Step 6. Dilate your plaques to ensure they are counted as single plaques
Select any value (0-31).

Step 7. Cut overlapping plaques so they are counted separately
Select any value (1-5).

Step 8.1. Define the minimum pixel size to count as a plaque
Select any value 0 to 1000.

Step 8.2. Define the maximum pixel size to count as a plaque
Select any value (0-10000).

Step 1. Select light setting

The Viridot counter analyzes pixel intensity values ranging from 0-1, requiring conversion of the raw image data into a single channel that can be analyzed as a greyscale image. The amount and type of light used for taking the well image determines the optimal color channel that provides the most information on the difference between background and plaque. The user can select the red, green, or blue channels, saturation, hue, or value channels (rgb2hsv() function in the grDevices package), or a combination of these channels.

The goal of the user is to find the light setting for which the plaques are white and the background is black. If your plaques appear dark and the background appears white, you can try the '1-...' options.

The following choices are available:

- hue
- value
- saturation
- red
- green
- blue
- 1-saturation
- 1-red
- 1-blue
- 1-green
- red and green only
- red and blue only
- blue and green only
- 1-(red and green only)
- 1-(red and blue only)
- 1-(blue and green only)

Notes:

- Play around with a few different light settings here, while you have the ***Show what is done at each step:*** box set to *Step 1. Select light setting*. Make sure you are seeing white plaques and black background. Find the light setting that makes the plaques lightest and background darkest. THIS STEP WILL AFFECT THE QUALITY OF YOUR PLAQUE COUNTING MORE THAN ANY OTHER STEP.
- Use of specific settings:
    - *saturation*: This setting is a choice for images where the plaques are a more 'saturated' or intense color than the background. For instance, this is what is used for dark blue plaques on a light blue background.
    - *1-saturation*: This setting is a good choice for images with dark lighting (e.g. brown background, blue-black plaques).
    - *1-red*: This setting is a choice for ELISPOT-like images, with reddish background and red/brown spots.
    - *1-green*: This setting is good for plaques with greenish/blue lighting.

Step 2. Blur image

This parameter tells Viridot how much you want it to blur/smooth the image. Images are smoothed to reduce noise by applying a Gaussian blur with sigma (radius in pixels for the Gaussian transformation) values range from 0 (no blur) to 5 (high blur). This is done to make it more likely that Viridot will recognize plaque-like pixels as a single object and not as a cluster of multiple plaques.

The following choices are available:

- The numbers are the sigma for the Gaussian blur applied to the image, and can range from 0 (no blur) to 5 (highly blurred).

Notes:

- If under the ***Plaque counting with automatic parameter identification?:*** box you selected *Auto*, this the blur variable will be automatically chosen based on analysis of your image.

Extra option: Remove strings/fibers in image

This parameter may work for some images, but not all. It tries to try to remove differently colored lines/strings on the plate. To remove non-plaque fibers that are a different color from plaques, *Extra option* is set to values >0 and transforms the image as: ***Extra option parameter value*** + *Step 1* + (1-blurred hue channel of image). ***Step 2. Blur Image*** is skipped if **Extra option: Remove strings/fibers in image** is used.

The following choices are available:

- Values range from 0 to 1.

Notes:

- This option is not used when ***Plaque counting with automatic parameter identification?*** is set to *Auto*.
- Higher values should do a better job removing lines (e.g. 0.4) but it will depend on your light settings and the color of your plaques.
- A value of 0 means that you are not using the ***Extra option: Remove strings/fibers in image***.

Step 3.1. Cut well edges

This parameter tells Viridot how many pixels you want to cut off the edge of the image so that the edge of the well is removed from the analysis. Images are trimmed so that only pixels more than a certain distance from the image edge are included in the analysis within a circle of radius ½(image height)-*Step 3.1*. This allows for exclusion of the well edges, which may contain reflections, excess liquid, and other distortions that can interfere with the plaque counting.

The following choices are available:

- Values range from 0 pixels cut to a maximum of 300 pixels. The default is value is 25.

Notes:

- If do not want to cut off any of the edge, set this to 0.

- If you want to cut off a lot of the well edge, set it to higher values.

Step 3.2. Insert value for pixels outside well

This parameter is a way of helping prevent the later thresholding steps from counting the well edge as plaques, rather than the actual plaques in the well. Filling in pixel values outside the well with the median value of background pixels improves later thresholding by reducing the likelihood that background is counted as plaques at the well edge (this especially important for well images with dark backgrounds).

The following choices are available:

- *Median background pixel value*: the pixels outside the well are set to the median value of the background pixels in each image.
- *Black (0):* the pixels outside the well area are set to 0.

Notes:

- If you have a lot background (e.g. grey background, rather than black), set this to *Median background pixel value*.
- If you do not have a high background, leave it at the default value of *Black (0)*
- If under the ***Plaque counting with automatic parameter identification?:*** box you selected *Auto*, this parameter will be automatically chosen based on analysis of your image.

Step 4.1. Apply contrast to image based on background intensity

This parameter creates a constant to multiply by the pixel values to increase the difference between plaque and background so that the thresholding step has an easier time distinguishing plaque from background. Pixel data are transformed to increase the difference between background and plaque with the equation: $Image_{transformed} = (\textbf{Step 4.1}) * Image^{(\textbf{Step 4.2})}$ (note: pixel values >1 are set to 1, and values <0 are set to 0). Image transformation helps ensure that the thresholding function specified in ***Steps 5.1*** and ***5.2*** is applied to the pixel difference between background and plaque, and not in some other minimum. Setting ***Step 4.1*** and ***Step 4.2*** to 1 skips this transformation step.

The following choices are available:

- Values range from 0.5 to 3.

Notes:

- Set this to a low value (eg. 0.5) if you have dark background.
- Set this to a high value (eg. 3) if you have light background.
- A value of 1 applies no contrast to the image.
- If under the ***Plaque counting with automatic parameter identification?:*** box you selected *Auto*, this parameter will be automatically chosen based on analysis of your image.

Step 4.2. Apply contrast to image based on plaque intensity

Like step ***Step 4.1***, this parameter is used to increase the difference between plaque and background so that the thresholding step has an easier time distinguishing plaque from background. This parameter exponentiates pixel values in the equation: $Image_{transformed}=(Step\ 4.1)*Image^{(Step\ 4.2)}$. (Note: pixel values >1 are set to 1, and values <0 are set to 0). Image transformation helps ensure that the thresholding function specified in ***Steps 5.1*** and ***5.2*** is applied to the pixel difference between background and plaque, and not in some other minimum. Setting ***Step 4.1*** and ***Step 4.2*** to 1 skips this transformation step.

The following choices are available:

- Values range from 1 to 4.

Notes:

- Set this to a low value (eg. 2) if you have smaller or paler plaques.
- Set this to a high value (eg. 3 or 4) if you have larger or darker plaques.
- A value of 1 applies no contrast to the image.
- If under the ***Plaque counting with automatic parameter identification?:*** box you selected *Auto*, this parameter will be automatically chosen based on analysis of your image.

Step 5.1. Select difference in pixel value to distinguish plaque from background

This parameter tells Viridot the minimum difference in pixel values between plaques and background. A thresholding function (makeBrush() and thresh() [20]) is used to analyze $Image_{transformed}$ to identify plaque shapes. A square thresholding filter (of the pixel size selected in ***Step. 5.2***) is applied to $Image_{transformed}$ with a difference in pixel intensity value selected using ***Step 5.1***. Pale plaques are best analyzed with low differences in pixel intensity value (0.06) while images with high background require an intermediate value (0.10).

The following choices are available:

- Values from 0.01 to 0.50.

Notes:

- A small difference, eg. 0.02, means that there is almost no difference between plaque and background, as is the case for very pale plaques.
- A larger difference, such as 0.12, may be useful for darker plaques on dark background, to prevent accidentally counting background as plaques.
- The default value is 0.08.
- If under the ***Plaque counting with automatic parameter identification?:*** box you selected *Auto*, this parameter will be automatically chosen based on analysis

of your image.

Step 5.2. Select size (in pixels) of the window for applying the thresholding algorithm to the image

This parameter defines the size (in pixels) of the square window used for applying the thresholding function to the image.

The following choices are available:

- Values from 10 to 40.

Notes:

- Smaller values (e.g. 10) will create a small pixel window of the image, a good choice for small, pale plaques.
- Larger values (e.g. 40) apply a larger pixel window in the image, and are better for large, dark plaques.

Step 6. Dilate your plaques to ensure they are counted as single plaques

This parameter adds pixels to the edge of each plaque. Shapes from the thresholding step (***Steps 5.1 and 5.2***) are dilated (***Step 6*** specifies the number of pixels added to the periphery of each object, dilate() [20]) to consolidate close objects that may, during thresholding, have been broken into multiple small objects but which are actually one plaque. Dilation also prevents small plaques from being broken into many pieces when we run the next step, which divides overlapping plaques. Dilation can be turned off by setting the ***Step 6*** to 0. Holes within objects are also filled at this step (fillHull() [20]).

The following choices are available:

- Values from 0-31.

Notes:

- Low values (1) will only add 1 pixel at each point around the circumference of the plaque while high values (e.g. 31) will add 31 pixels at each point around the circumference make the plaques look round and big.
- The default is 11.
- This is an important step to consider if you want to measure the true size of your plaques.

Step 7. Cut overlapping plaques so they are counted separately

This tells the Viridot how eager it should be in dividing up objects into separate plaques. A watershed function (distmap() and watershed() [20]) is applied so that distinct plaques that are touching are counted as separate objects. The user can specify the tolerance

for touching objects, with low values (1) dividing more objects and higher values (3 as default, 5 as high) for dividing fewer objects.

The following choices are available:
- Values range from 1 to 5.

Notes:
- Small values (1) will draw more lines while high values (5) will almost never divide objects into multiple plaques.
- The default setting is 3.

Step 8.1. Define the minimum pixel size to count as a plaque

This step tells the program the smallest number of contiguous pixels (plaque 'area') to count as a plaque. The user specifies the minimum size of an object (total number of pixels/object) that should be counted as a plaque.

The following choices are available:
- Values range from 0 to 1000.

Note:
- The default is 20.

Step 8.2. Define the maximum pixel size to count as a plaque

This step tells the program the largest number of contiguous pixels (plaque 'area') to count. The user specifies the maximum size of an object (total number of pixels/object) that should be counted as a plaque.

The following choices are available:
- Values range from 0 to 10000.

Note:
- The default is 10000.

4. Neutralization titer estimation tab

4.1. Load files and plot data (CENTER PANEL, FIRST TAB)

Select the directory that contains your plaque count data

To load a plate's worth of plaque count data to analyze, press the button ***Select the directory that contains your plaque count data***. Within that window, click on the folder containing tables of plaque data and press ***Select***.

Select the table/tables of plaque counts

In the box below the title ***Select the table/tables of plaque counts***, the files listed in the previous folder selected with the button ***Select the directory that contains your plaque count data*** should appear. Choose one or more tables of plaque data.

Make sure the dimensions of the tables you have imported match the dimensions of the template (shown in the ***Template and tables*** tab). For instance, if you want to read in a table of 8 rows x 12 columns (plus row and column names), you need a template that is also 8 rows x 12 columns (plus row and column names). Make sure the table of plaque data table is oriented in the same way as the template. You can also read in 24 well plate data, or any size table you want, but again you must make sure your template matches the plate dimensions. Otherwise, you will get an error message.

Select template

Viridot will analyze your data based on the template you use (the current default is set to template shown in the section ***Neutralization titer estimation example***). To ensure the table and template match, you can define a well as a sample of a certain dilution (e.g. S1-D1) or as a control (e.g. C1, C2, etc.). Viridot will automatically generate summary statistics (mean, median, etc.) for wells with the same label in the format *C#*, e.g. *C1*, *C2*, etc. in the template. These names (e.g. *D11,D12* or *C1,C2* etc.) can also be entered in the box ***Number of plaques in control wells*** to define the plaque count of controls.

The default template assumes you have imported a 96-well plate. In this template, there are four different samples (S1, S2, S3, and S4) each with 12 dilutions (D1, D2, D3, D4, D5, D6, D7, D8, D9, D10, D11, D12) and each sample is done in duplicate (there are two wells on each plate entitled S1-D1, etc.).

You can design your own template by making a copy of the template files in the folder Example_templates_of_plates_for_neutralization_titer_estimation and modifying them to your own needs.

To change the template, click the tab ***Load files and plot data*** and press the ***Select template*** button.

Estimate neut. titers

Press this button to estimate the neutralization titers for the selected plate/plates of data.

Warning messages appear here:

Any error and warning messages will appear here.

4.2. Template and tables (CENTER PANEL, SECOND TAB)

The imported or default template (*template_96_well_plate_default.csv*) as well as the first plaque count table you have imported will appear in this tab, either after you press the ***Estimate neut. titers*** button or after pressing the ***Select template*** button.

4.3. Saving neutralization titers (RIGHT PANEL)

Save neutralization curves and data?

Here, you indicate whether you would like the neutralization titer curves and data to be saved when you press the ***Estimate neut. titers*** button. If you say *Yes*, but have not selected an output directory using the button ***Select the directory for saving neutralization data***, you will get the warning message: *You need to select an output directory using the button 'Please select a directory for saving neutralization data output'. If you do not want to save the neutralization curves and data say No*.

Select the directory for saving neutralization data

Here, you select a directory where you would like the neutralization curves and neutralization titers to be saved. Make sure this is NOT the same as where your well images are saved. Once you have selected the directory, press the ***Estimate neut. titers*** button. Two files will be created in this directory: both will include the name of the plate you imported (e.g. *awesome-plaque-data*) and the name provided in the box ***Experiment name*** (default is: *unique-name*). The neutralization titer data will be printed into a file with the ending *–data.xls* and the image file of the neutralization curves in the file ending in *.png*. For these examples, the two files would be named: *awesome-plaque-data-unique-name-data.xls* and *awesome-plaque-data-unique-name.png*.

4.4. Provide names for samples and experiment (LEFT PANEL)

Sample names

You can change the sample/serum names to be whatever you like. For instance, *SR-*

1,SR-2,SR-3,SR-4 can become *Patient 1,Patient 2,Patient 3,Patient 4*. In the figure and output data tables, the title (SR=) should change to contain the sample names you input. Critically, however, the number of sample names in this list must match the number of samples (S1 S2 S3 etc.) indicated in your TEMPLATE. If not, an error message will appear saying: 'The number of serum names given doesn't match the number of samples in your template. Please correct this.'

Default value is: *SR-1,SR-2,SR-3,SR-4.*

Virus names

You can also input your own virus names. If all samples indicated in ***Sample names*** box were titrated against the same virus, just put in one virus name, e.g. dengue virus. If you have two viruses in the ***Virus names*** box but three distinct sample names, only the first virus name input will be printed on the plot and in the output data tables. However, if the number of virus names matches the number of sample names, all virus names will be printed on the plot and in the output data tables. For instance, if Patient 1's sample was titrated against dengue virus, Patient 2's against Zika virus, and Patient 3's against chikungunya virus, it would show as follows:

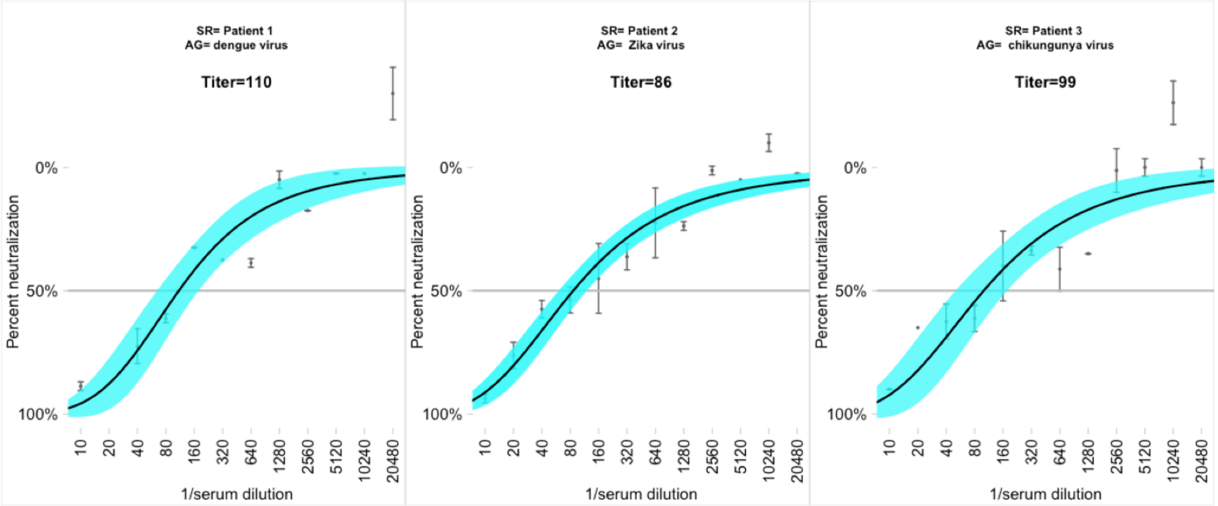

Default value is: *Virus-1*.

Experiment name

Here, you can write in a unique experiment name for your table. See section ***Select the directory for saving neutralization data*** for more details.

The default value is: *unique-name*.

Run only a subset of samples

Perhaps you only want to run certain subsets of the samples on your plate, e.g. S1 and S3, but not S2 and S4. You can specify that here by listing the samples you want plotted (e.g. S1,S3).

Default value is: *blank*.

4.5. Provide information on dilution series and neutralization titer estimation (LEFT PANEL)

Starting serum/antibody dilution

Here, you input the starting dilution for your neutralization assay. For instance, if you started with a serum dilution of 1:10 you would put 10. If you are measuring the neutralization of a monoclonal antibody, you could put 5000, indicating a starting concentration of 5000 ng/mL. Make sure the number of dilutions printed after pressing the button ***Show serum dilutions selected*** box matches the number of dilutions you indicated in your template.

Default value is: *10*.

Dilution series (e.g. 2=2-fold, 3=3-fold, 4=4-fold)

Here, you indicate the dilution series you have done: 2, for 2-fold serial dilutions, 3 for 3-fold serial dilutions, etc. Make sure the number of dilutions printed after pressing the button ***Show serum dilutions selected*** box matches the number of dilutions you indicated in your template.

Default value is: *2*.

Final serum/antibody dilution

Here, you input the final dilution for your neutralization assay. For instance, if your final serum dilution were 1:20480, you would put 20480. If you are measuring the neutralization of a monoclonal antibody, you could put 2.44, indicating a final antibody concentration of 2.44 ng/mL. Make sure the number of dilutions printed after pressing the button ***Show serum dilutions selected*** box matches the number of dilutions you indicated in your template.

Default value is: *20480*.

Number of decimal places to print for titers/CI/slope

Number of decimal places to print for titers/CI/slope: Here, you can indicate whether you want your titers/CI/slope rounded with no decimal places (0) or with some number of decimal places (e.g. 2). Make sure to specify a value greater than 0 if you are studying monoclonal antibodies at concentrations less than 1. Slope printed as this value plus two decimal places.

Default value is: *0*.

X-axis label

Here, you can input the relevant X-axis label for your plot, depending on whether you are titrating a serum or an antibody.

Default value is: *1/serum dilution*.

Show serum dilutions selected

Press this button to see dilution series you created with the selections in boxes ***Starting serum/antibody dilution, Dilution series (e.g. 2=2-fold, 3=3-fold, 4=4-fold)***, and ***Final serum/antibody dilution***. Make sure the number of dilutions printed in the first column matches the number of dilutions you indicated in your template.

Default value is:

1	10
2	20
3	40
4	80
5	160
6	320
7	640
8	1280
9	2560
10	5120
11	10240
12	20480

Lower titer limit

The limit of your neutralization curve may be different from your assay limit of detection. For instance, you may want to estimate the $PRNT_{50}$ value down to a serum dilution of 1:5, but you only titrated antisera with a starting dilution of 1:10. You can put serum

dilutions/antibody concentrations within or outside (maximum of 32-fold difference) of the range indicated in ***Show serum dilutions selected***.

Default value is: *10.*

Upper titer limit

The limit of your neutralization curve may be different from your assay limit of detection. For instance, you may want to estimate the $PRNT_{50}$ value above the final serum dilution indicated, e.g. 1:40960. You can put serum dilutions/antibody concentrations within or outside (maximum of 32-fold difference) of the range indicated in ***Show serum dilutions selected***.

The default value is: *20480*.

LID Statistical Web Tools PRNT generator method?

Some researchers use the LID Statistical Web Tools plaque reduction tool: https://exon.niaid.nih.gov/plaquereduction/ (details on this method are provided on the website by clicking beneath the title LID Statistical Web Tools on that website). We provide an approximation of this tool. This can be turned on by selecting TRUE.

The default value is: *FALSE*.

Allow resistant fraction?

This setting allows the user to estimate a resistant fraction for the neutralization curve. Not all antibodies or antisera achieve 100% plaque reduction; some only ever achieve e.g. 70% plaque reduction even at saturating concentrations of antibody. Selecting TRUE will estimate a curve with a lower asymptote, FALSE will force the curve to span from 100% to 0% plaque reduction.

Default value is: *FALSE*.

Percent plaque reduction

Here, you can select the desired percent plaque reduction value to report. Values from $PRNT_{10}$ to $PRNT_{90}$ are allowed.

The default value is: $PRNT_{50}$.

Number of plaques in control wells

The box ***Number of plaques in control wells*** there are multiple choices for defining plaque counts for control wells:

- *Eachtop* estimates the upper asymptote of the curve (using a 4-parameter logistic regression) to estimate the control well plaque count. If this fails, the median value for the last three dilutions for each neutralization titration is used.
- A number (e.g. *45*) input by the user, possibly corresponding to a relevant mean of plaque counts in a control well.
- A reference to the template, e.g. if *C1* is written, the control will be estimated from the median of counts in wells labeled *C1* in the template. The user can also input multiple wells here, e.g. *C1,C2* if desired, and a median will be estimated for all wells with those labels. The reference can also be to a specific dilution, e.g. *D10* or *D11,D12*, etc. The program will not recognize template designations based on *S* (meaning you cannot say S1 and have it analyze the median for that sample—you would need to rename that sample C1 for the program to be able to estimate the median for those wells).

The default value is: *D10,D11,D12*, meaning the top three dilutions for all wells on the plate.

4.6. Choices for plotting neutralization curves

Color of confidence interval

Choose the color of the confidence interval. All named colors in R are available for selection. The transparency of the confidence interval is related to the width of the confidence interval—narrower confidence intervals are darker, wider confidence intervals are lighter.

Default value is: *cyan*.

Color of regression line

Choose the color of the regression line. All named colors in R are available for selection.

Default value is: *black*.

Plot duplicate values

Here, you can select how to display your raw plaque count data on the figure. You can

select *error bars (standard deviation),* which estimates the standard deviation of repeats, or *polygon*, which plots the area between the highest and lowest counts for each dilution.

Default value is: *error bars (standard deviation).*

5. Other virology tools tab

5.1. Estimate dilution needed to achieve a certain number of plaques per well for neutralization experiments

Titer of initial virus stock (X, as 10^X)

This is the $\log_{10}$ of the number of plaque forming units (pfu)/mL in your virus stock (possibly as estimated with the ***Estimate virus titer from a virus titration*** program, below). For instance, if you had 26302680 pfu/mL that would be $\log_{10}(26302680)=7.42$ (which is the default value).

Target number of plaques per well in experiment

This is the number of plaque forming units you want to observe in each well. Default value is 40 pfu/well.

Final volume of diluted virus required for full experiment (in µL)

This number is the final volume, in µl, of diluted virus that you need for your experiment. The default value is 5000 (if, for instance, you needed to add 40µL to each well on a 96 well plate, you would need 4800µL, which rounded up for volume loss could be 5000µL).

Volume of virus added to wells of cells in experiment (in µL) (* Virus only, not total serum+virus mixture)

This is the total volume of virus that will be added to the cells (not the amount you prepare as a serum+virus mixture). Default value is 15µL.

Estimate virus dilution

The virus dilution you should do for your experiment, based on the inputs specified above, will be printed when you press this button.

5.2. Estimate virus titer from a virus titration

Starting dilution of virus titration (first row on plate, e.g. 1/10)

This is where you indicate the starting dilution of your virus sample. Default is 0.01 (1/10).

What dilution series did you use for your virus titration (e.g. 10-fold)

This is where you indicate the dilution series used for your virus titrations. The default is 10-fold serial dilutions.

Which row on plate (which dilution number) are the plaque counts input here from?

Here, you indicate from which well in the titration you acquired the plaque counts input in the box ***Number of plaques (can input multiple numbers separated by a comma) per well counted***. The default is 5, meaning, the 5th well in the titration.

Volume transferred to wells of cells in virus titration (in µL)

This is the total volume of virus that was added to the cells. The default value is 30µL.

Number of plaques (can input multiple numbers separated by a comma) per well counted

Here, you input the number of plaques you observed for the well/wells indicated in the box ***Which row on plate (which dilution number) are the plaque counts input here from?***. You can put a single value here or multiple values separated by commas. The program will automatically estimate the titer from the mean of these counts. The default is 40,44, and 50 plaques/well.

Estimate virus titer

The virus titer from your virus titration, based on the inputs specified above, will be printed when you press this button.

6. Image formatting

Directory of images to be trimmed

Click this button to select the directory that contains either the plate you would like to cut into individual well images or the individual well images you would like to cut into squares.

Select the well images to trim

Here you should select all of the plate images or well images you would like to trim. A list of images to choose from will appear after you select the directory in the previous step.

What do you want to do?

Here you can choose to either (1) trim individual well images into squares for running in Viridot or (2) cut a single large image into individual square images for running in Viridot.

Directory for saving trimmed images

Click this button to select the directory where you would like to save your trimmed images.

Print out individual square wells from single large image?

This step only applies if you chose to "Cut single large image into individual square wells". Choose here whether or not you want to output the trimmed images at this time. You may choose no, for instance, if you are optimizing the parameters for how to correctly trim plate images into square well images to make sure wells are centered in the middle of each well image. When you press trim, you will be shown how the grid is applied to the plate image.

How many pixels per well?

This step only applies if you chose to "Cut single large image into individual square wells". Here you specify the dimensions, in pixels, for each well. You can play around with this parameter to figure out how large each well image should be.

How far from the left of the image should the grid start?

This step only applies if you chose to "Cut single large image into individual square

wells". This parameter is used to shift the grid applied to a plate image to trim it into individual wells. This will shift the grid left and right.

How far from the top of the image should the grid start?

This step only applies if you chose to "Cut single large image into individual square wells". This parameter is used to shift the grid applied to a plate image to trim it into individual wells. This will shift the grid up and down.

Trim!

Press trim to run the specified program.
