# Supplementary material for: Viridot: An automated virus plaque (immunofocus) counter for the measurement of serological neutralizing responses with application to dengue virus
Source: PLoS Negl Trop Dis. 2018 Oct 24;12(10):e0006862. doi: 10.1371/journal.pntd.0006862 (PMC6226209; doi:10.1371/journal.pntd.0006862)
Supplement: S2 Supporting File — (PDF) [file pntd.0006862.s007.pdf]

Plaque reduction neutralization test and image acquisition protocol for dengue virus (DENV) on C6/36 cells

C6/36 cells (*Aedes albopictus*, NIAID/Novavax MCB, passage 9-22) were seeded at $10^{4.5}$ cells/well and allowed to divide until wells were confluent (1 day) with 200µl culture medium. Serum aliquots (from either de-identified biorepository samples from prior clinical trials of monovalent components of a live attenuated dengue vaccine [24] or samples from non-human primates each inoculated with a distinct DENV strain [25]) were rapidly thawed, heat inactivated in a water bath at 56°C for 30 minutes, then diluted 1:10 in diluent (OptiMEM, 2% FBS, 50 µg/mL gentamicin, and 0.5% albumin). Twelve two-fold serial dilutions of antisera (1:10 to 1:20,480 with 0.04 mL per well) were prepared in 96 well plates. DENV samples [24,26] were diluted to yield an expected count of 40 plaques per well, and 0.04 mL of the diluted virus samples were added to the serum dilutions. Although the dilution of the antiserum in the final virus-serum mixture ranges from 1:20 to 1:40,960, we report serum dilution prior to mixing with virus (1:10 to 1:20,480). Plates with diluted serum and virus were incubated for 90 minutes at 37°C, 5% CO_2, 80% relative humidity. Aliquots of the serum-virus mixture (0.03 mL) were added in duplicates to 96-well plates of confluent C6/36 cells from which culture medium was removed and incubated for 1 hour at 32°C. After incubation, 0.150 mL warmed overlay medium (OptiMEM GlutaMAX, 1% methylcellulose, 2% FBS, 2.5 µg/mL amphotericin B, 20 µg/mL ciprofloxicin, at 37°C) was added to all wells, and plates were incubated for 3 days at 32°C, 5% CO_2 and 90% humidity.

Plates were removed from the incubator and overlay medium was discarded. Plates were washed with phosphate buffered saline (PBS buffer), and fixed at room temperature for 30 minutes with 0.1 mL of 80% methanol. Methanol was removed, and an antibody dilution buffer, made with 1X PBS and blocking agent 5% weight/volume non-fat dry milk, was added to all wells and left for 10 minutes. Flavivirus mouse monoclonal antibody 2H2 (HB112 hybridoma from ATCC, 0.8 mg/mL) was diluted 1:2000 in antibody dilution buffer and 0.1 mL was added to each well. Plates with the primary antibody solution were shaken (~60 rpm) at 37°C for 2 hours. Primary antibody solution was removed, and plates were washed twice using an automated plate washer with 0.18 mL PBS. Peroxidase-labeled goat anti-mouse IgG (Kirkegaard & Perry Laboratories, KPL, Gaithersburg, MD) was diluted 1:3000 in antibody dilution buffer, 0.1 mL was added to each well, and plates were shaken gently at 37°C for 1 hour. The secondary antibody solution was removed and plates were washed twice using an automated plate washer with PBS. Plates were allowed to dry before adding 0.03 mL TrueBlue Peroxidase substrate (KPL), which was left on wells until plaques were clearly distinguishable (5-40 minutes). The TrueBlue substrate was then removed.

Plates were scanned using the ImmunoSpot Analyzer (CTL, Shaker Heights, OH) set to the Costar 96 well template. The plate and light settings for the Costar 96 well template are shown in S1 Table. Well images were saved as .CTL images of 752 by 752 pixels.

Plaque reduction neutralization test and image acquisition protocol for DENV and Zika virus (ZIKV) on Vero cells

Vero '81 cells (CCL-81, ATCC, Manassas, VA, passage 142-149) were seeded at 20,000 cells/well in 100µL of culture medium and incubated overnight (18-22 hrs) at 37°C with 5% CO_2 and 80% relative humidity. To assess neutralization potential of serum, de-identified biorepository samples from a prior clinical trial of a live attenuated dengue vaccine [27] were diluted in six 4-fold increments ranging from 1:5 – 1:5,120 in a final volume of 0.06 mL. For assessment of neutralization potential of monoclonal antibodies (human anti-E dimer epitope [EDE] mAbs B7, C8, and C10 [28]), these were prepared in six five-fold serial dilutions with concentrations ranging from 1000 to 0.32 nL/mL. DENV and/or ZIKV samples were diluted to yield an expected count of 40-150 immunofoci per well, and 0.06 mL of the diluted virus samples were added to the antibody dilutions. Plates with diluted antibody and virus were incubated for 30 minutes at 37°C, 5% CO_2, 80% relative humidity. Aliquots of the antibody-virus mixture (0.03 mL) were added in duplicates to 96 well plates of confluent Vero cells, and incubated for 1 hour at 37°C with 5% CO_2 and 80% relative humidity. After incubation, approximately 0.100 mL warmed overlay medium (OptiMEM GlutaMAX, 1% methylcellulose, 2% FBS, 50 µg/mL gentamicin at 37°C) was added to all wells, and plates were incubated for 2 days at 37°C with 5% CO2 and 80% relative humidity.

Plates were removed from the incubator and overlay medium was discarded. Plates were washed with phosphate buffered saline (PBS) twice, and fixed at 4°C for 30 minutes with 0.1 mL of 50% methanol 50% acetone. 50:50 methanol:acetone was

removed, and then plates washed with PBS twice and an antibody dilution buffer, made with 1X PBS and blocking agent 5% weight/volume non-fat dry milk, was added to all wells and left for 10 minutes at room temperature (RT). 50µL primary antibody diluted in antibody dilution buffer added to each well (flavivirus mouse monoclonal antibodies 2H2 (ATCC HB-112, produced in bulk by Lofstrand, Gaithersburg, MD, 1.63 mg/mL) and/or 4G2 (ATCC HB-112, produced by Lofstrand, 0.8 mg/mL) both diluted 1:1000 in antibody dilution buffer). Plates with the primary antibody solution incubated at 37°C with 5% CO2 and 80% relative humidity for one hour. Primary antibody solution was removed and the plates were washed with PBS twice and 50uL secondary antibody added to each well (Peroxidase-labeled goat anti-mouse IgG [Kirkegaard & Perry Laboratories, KPL, Gaithersburg, MD] was diluted 1:2000 in antibody dilution buffer). Plates incubated at 37°C with 5% CO_2 and 80% relative humidity for one hour. The secondary antibody solution was removed and the plates were washed with PBS twice. 0.50 mL TrueBlue Peroxidase substrate (KPL) was added to wells until plaques were clearly distinguishable (1-2 minutes). The TrueBlue substrate was then removed and plates were washed once with 0.1mL ddH_2O.

Images of plates were generated using a Zeiss Axio Imager.M1 microscope with a 2.5X lens and the AxioCam MRc mounted camera controlled by the software KSElispot (Windows 95). Well images were saved as .TIF images of 1040 by 1040 pixels.
